# BosR (BB0647) Controls the RpoN-RpoS Regulatory Pathway and Virulence Expression in *Borrelia burgdorferi* by a Novel DNA-Binding Mechanism

**DOI:** 10.1371/journal.ppat.1001272

**Published:** 2011-02-10

**Authors:** Zhiming Ouyang, Ranjit K. Deka, Michael V. Norgard

**Affiliations:** Department of Microbiology, University of Texas Southwestern Medical Center, Dallas, Texas, United States of America; Medical College of Wisconsin, United States of America

## Abstract

In *Borrelia burgdorferi* (Bb), the Lyme disease spirochete, the alternative σ factor σ^54^ (RpoN) directly activates transcription of another alternative σ factor, σ^S^ (RpoS) which, in turn, controls the expression of virulence-associated membrane lipoproteins. As is customary in σ^54^-dependent gene control, a putative NtrC-like enhancer-binding protein, Rrp2, is required to activate the RpoN-RpoS pathway. However, recently it was found that *rpoS* transcription in Bb also requires another regulator, BosR, which was previously designated as a Fur or PerR homolog. Given this unexpected requirement for a second activator to promote σ^54^-dependent gene transcription, and the fact that regulatory mechanisms among similar species of pathogenic bacteria can be strain-specific, we sought to confirm the regulatory role of BosR in a second virulent strain (strain 297) of Bb. Indeed, BosR displayed the same influence over lipoprotein expression and mammalian infectivity for strain Bb 297 that were previously noted for Bb strain B31. We subsequently found that recombinant BosR (rBosR) bound to the *rpoS* gene at three distinct sites, and that binding occurred despite the absence of consensus Fur or Per boxes. This led to the identification of a novel direct repeat sequence (TAAATTAAAT) critical for rBosR binding *in vitro*. Mutations in the repeat sequence markedly inhibited or abolished rBosR binding. Taken together, our studies provide new mechanistic insights into how BosR likely acts directly on *rpoS* as a positive transcriptional activator. Additional novelty is engendered by the facts that, although BosR is a Fur or PerR homolog and it contains zinc (like Fur and PerR), it has other unique features that clearly set it apart from these other regulators. Our findings also have broader implications regarding a previously unappreciated layer of control that can be involved in σ^54^–dependent gene regulation in bacteria.

## Author Summary

Lyme disease, caused by the bacterium *Borrelia burgdorferi* (Bb), remains the most common arthropod-borne illness in the United States. A critical strategy for Bb to maintain its presence in nature is adaptation to its diverse tick and mammalian (mouse) hosts. To accomplish this, Bb encodes a potential gene regulator, BB0647 (BosR). Herein, we confirmed that BosR is essential for Bb to establish mammalian infection. We then found that purified recombinant BosR bound to the promoter DNA (regulatory region) of *rpoS*, suggesting that BosR directly controls the expression of the *rpoS* gene. This study has revealed a new mechanism of bacterial gene control. The discovery that BosR governs Bb's virulence may lead to new strategies to interrupt the bacterium's complex life cycle.

## Introduction

Bacterial gene expression is primarily controlled at the transcriptional level, which requires a central DNA-dependent RNA polymerase (RNAP) consisting of catalytic core (α_2_ββ'ω; E) and a dissociable σ factor [1]. Gene transcription occurs when the Eσ-promoter closed complex (CC) is converted to the open complex (OC). Among various σ factors, the alternative σ factor σ^54^(σ^N^, RpoN) is employed by many bacteria to transcribe genes involved in a wide variety of cellular functions such as virulence, nitrogen metabolism, and stress responses [1]. Unlike other σ factors, the Eσ^54^ holoenzyme alone cannot melt the promoter. The Eσ^54^-CC (held by the interaction of Eσ^54^ with the unique -24/-12 promoter) remains in this conformation until the activator ATPase interacts with RNAP, which hydrolyzes ATP for promoter melting [2]–[4]. The activator ATPase, also known as the enhancer-binding protein (EBP), usually binds to an enhancer site located ∼100–150 bp upstream of the promoter. Typically, the EBP interacts with the RNAP via a DNA looping mechanism that is modulated by a DNA-bending protein such as integration host factor (IHF).

*Borrelia burgdorferi* (Bb), the Lyme disease spirochete [5]–[6], encodes three σ factors: the housekeeping σ^70^ (RpoD, BB0712), and two alternative σ factors, σ^54^ (RpoN, BB0450) and σ^S^ (RpoS, BB0771) [7]. Abundant evidence [8]–[11] has revealed that Bb RpoN directly binds to the -24/-12 site in *rpoS* promoter and thus activates *rpoS* which, in turn, modulates the differential expression of more than 100 genes involved in Bb virulence, stress adaptation, and many other functions. Studies have indicated that the Bb RpoS regulon is triggered by various environmental stimuli including temperature, pH, cell density, and unknown mammalian host factors [12]–[15]. The RpoN-RpoS pathway (or the Rrp2-RpoN-RpoS pathway) [8]–[11], [16]–[18] plays a central role in modulating the differential expression of Bb outer surface lipoproteins such as outer surface protein C (OspC) [14], [19]–[20] and decorin-binding protein A (DbpA) [21]–[23], which are critical for Bb to transmit from the arthropod tick vector to mammalian hosts and to maintain its natural life cycle. Activation of the RpoN-dependent *rpoS* gene requires the activation of Rrp2 (BB0763), a putative EBP [17]. Although Rrp2 was presumed to be the sole NtrC-like EBP in Bb, Rrp2 seems to be unconventional as an EBP in that it apparently does not bind specifically to the RpoN-dependent promoter of *rpoS* [8], [24]. The efficient translation of *rpoS* mRNA also requires the small RNA DsrA and an atypical RNA chaperone Hfq [25]–[26].

Emanating from our general interest in virulence expression in the Lyme disease spirochete, we previously showed that a manganese transporter, BB0219 (BmtA), is required for full virulence by Bb [27]. The implication that metal transport, and perhaps metal control over borrelial gene regulation, could influence Bb's virulence prompted us to expand our study to examine other molecules of Bb implicated in metal-sensing. To this end, BosR (BB0647) is a ferric uptake regulator (Fur)-like homologue in Bb [7], [28] that has been postulated to contribute to the regulation of oxidative stress responses [29]. In many bacteria, Fur globally regulates iron homeostasis and other functions [30]. Given the provocative finding that Bb seems not to accumulate iron [31], it remained tenuous as to whether BosR is involved in iron uptake by Bb. Nonetheless, BosR may influence cellular functions other than iron acquisition. Recently, we and others surprisingly found that expression of the RpoS regulon was significantly impaired in a mutant deficient in *bosR*, leading to the additional unexpected finding that BosR somehow functions as a second activator to promote σ^54^-dependent *rpoS* transcription, and that such control by BosR ultimately governs the expression of virulence-associated membrane lipoproteins and mammalian infectivity by Bb [32]–[35]. Although this discovery has represented a major advance in further understanding the regulatory control of virulence expression by the Lyme disease spirochete, the observation has engendered many new unanswered questions. Among them include whether BosR is indeed a global regulator common to more than the one virulent strain of Bb (examined in previous studies) [32]–[34], and how BosR may act mechanistically to exert its positive control over the RpoN-RpoS regulatory pathway in Bb. In this report, we provide further evidence for the direct involvement of BosR in the activation of *rpoS*, and thus the RpoS regulon, in a second virulent strain of Bb. We also present evidence that BosR functions as a DNA-binding protein, but it has many features that markedly distinguish it from either of its Fur or PerR homologs. Defining this novel involvement of BosR relative to its control over the RpoN-RpoS pathway is important for elucidating Bb's host adaptation and pathogenesis, and could lead to innovative strategies for thwarting Lyme disease. This study also expands our understanding of bacterial sigma factor regulatory networks, and establishes a new paradigm of an additional transcriptional activator that is absolutely required for σ^54^–dependent gene regulation in a bacterial pathogen.

## Results

### Inactivation of *bosR* in Bb strain 297

Previously, we [34] and others [32] found that the mutation of *bosR* in Bb strain B31 abolished RpoS, OspC and DbpA expression. However, there are some notable discrepancies between these two studies. Hyde *et al.* [32] found that the mutant exhibited defects in growth *in vitro* and in the expression of NapA (or Dps, implicated in protecting DNA from damage during starvation or oxidative stress), whereas we [34] showed that the *bosR* mutant had normal growth and NapA expression comparable to WT Bb. It is also well-documented that transcriptional regulators and gene control mechanisms can differ widely among bacterial pathogens of the same species [36]–[38], and variations in strain-specific genetic contents, gene expression profiling, and pathogenicity have been observed, in particular, for different strains of Bb [39]–[40]. Thus, to more broadly investigate the role of BosR in Bb pathogenesis and gene regulation, we generated *bosR* mutants in another virulent WT Bb strain (strain 297) via homologous recombination. As in strain B31 [34], *bosR* in strain 297 was predicted to be cotranscribed and form an operon with *bb0646* and *bb0648* (Fig. S1A). To verify this, RT-PCR using specific primers and Bb cDNA was performed. As shown in Fig. S1B, amplicons spanning the junction of *bb0646*/*bosR* (lane 3), *bosR/bb0648* (lane 5), or *bb0646- bb0648* (lane 6) were generated, indicating the operonic nature of *bb0646*, *bosR*, and *bb0648*. Of note, the sequences of this operon and its flanking genes (*bb0645* and *bb0649*) are identical between both strains 297 and B31 (data not shown). Thus, the same strategies used in the creation of the *bosR* mutant in B31 [34] were employed to inactivate *bosR* in Bb 297. When the suicide vector pOY24 was transformed into Bb 297, two kanamycin-resistant *bosR* mutant clones (OY08/A11 and OY08/F4) were obtained. To *cis*-complement the *bosR* mutation, the suicide plasmid pOY83 [34] containing the *bb0649*-*bb0648*-*bosR*-P*flgB*-*aadA* cassette was introduced into the *bosR* mutants. As a result, two streptomycin-resistant clones (OY33/A6 and OY33/F7) were isolated. The inactivation and complementation of *bosR* in these strains were confirmed using PCR (Fig. S1C). Moreover, RT-PCR and immunoblot analyses revealed that BosR expression was detected in both WT and the complemented strains, but not in the mutants (Fig. S1D and E).

To ensure that all mutants and complements retained the plasmids cp26, lp25 and lp28-1 that are essential for Bb virulence [41]–[42], PCR-based plasmid profiling was performed. As shown in Fig. S2A, the WT and *bosR* mutant OY08/A11 contained the same plasmid profiles. In addition, OY08/F4 and the complemented strains (OY33/A6 and OY33/F7) contained the same plasmid profiles as that of the WT strain (data not shown). The *bosR* mutant exhibited spirochetal morphology and movement identical to that of WT under dark-field microscopy. No discernable growth defect was observed when *bosR* was inactivated, and the mutants displayed similar growth patterns to that of WT (Fig. S2B).

### BosR is essential for Bb to establish mammalian infection

The role of BosR in Bb strain 297 mammalian infectivity was assessed using the murine needle-challenge model of Lyme borreliosis [43]–[44]. All mice inoculated with WT or the complemented strains at a 10^4^ inoculum became infected, and motile spirochetes were isolated from all tissues from these mice (Table 1). In contrast, the *bosR* mutants were not recovered from any mice inoculated with either 10^4^ or 10^7^ bacteria. These data establish that previous findings implicating BosR in Bb's infectivity and virulence [32], [34] were not unique to strain B31, and thus BosR appears to be essential for conferring virulence to other pathogenic strains of Bb.

**Table 1:** Infectivity of Bb clones in mice.

Strain, clone	Description	Dose	No. of cultures positive/total No. of specimens examined				No. of mice infected/total No. of mice
			Heart	Joint	Skin	All sites	
297	wild-type Bb	10^4^	9/9	9/9	9/9	27/27	9/9
OY08/A11	*bosR* mutant	10^4^	0/6	0/6	0/6	0/18	0/6
OY08/F4	*bosR* mutant	10^4^	0/7	0/7	0/7	0/21	0/7
OY08/A11	*bosR* mutant	10^7^	0/10	0/10	0/10	0/30	0/10
OY08/F4	*bosR* mutant	10^7^	0/6	0/6	0/6	0/18	0/6
OY33/A6	complement	10^4^	6/6	6/6	6/6	18/18	6/6
OY33/F7	complement	10^4^	6/6	6/6	6/6	18/18	6/6

Data were collected from three independent experiments.

### BosR controls the expression of *rpoS*, *ospC* and *dbpA*

**Figure 1:**
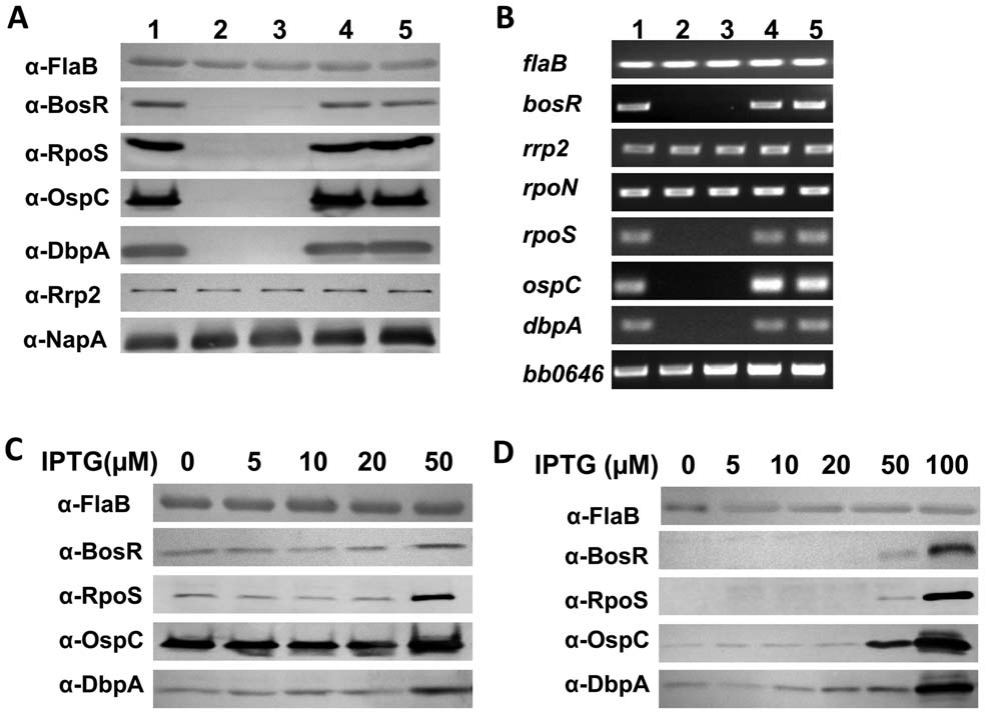
BosR activates the expression of *rpoS*, *ospC* and *dbpA*. (A, B) Gene expression in spirochetes grown in BSK-H medium (pH 6.8) at 37°C was assessed by immunoblot (A) and RT-PCR (B). Lane 1, WT 297; lane 2, *bosR* mutant OY08/A11; lane 3, *bosR* mutant OY08/F4; lane 4, complement OY33/A6; lane 5, complement OY33/F7. (C) Induction of *bosR* by IPTG leads to increased production of RpoS, OspC and DbpA. WT 297 containing the IPTG-inducible *bosR* construct (pOY112) was grown with various concentrations of IPTG. Cells were harvested at late-log phase and analyzed by immunoblot. (D) Complementation of *bosR* mutation in *trans* rescues gene expression. The *bosR* mutant OY08/A11 harboring pOY112 was grown with varying IPTG. Cells were analyzed by immunoblot. Specific antibodies, indicated as α-, used in the immunoblot are indicated on the left. FlaB was used as a normalization control for equivalent loading.

To determine whether the loss of Bb strain 297 virulence in the *bosR* mutant correlated with a loss in the expression of *rpoS*, *ospC*, and *dbpA*, as has been noted for strain B31 [32], [34], we further assessed the effect of the *bosR* mutation on gene expression. WT 297, the *bosR* mutants, and the *cis*-complemented strains were cultured at 37°C in BSK-H medium at pH 6.8, conditions under which the RpoS regulon is highly induced [10], [14]–[15], [25]. Cells were harvested at late-log phase and subjected to immunoblot and RT-PCR analyses. As shown in Fig. 1, when *bosR* was inactivated, the expression of *rpoS*, *ospC* and *dbpA*, was essentially abolished at both the protein (Fig. 1A) and mRNA (Fig. 1B) levels. When the *bosR* mutation was *cis*-complemented, gene expression was fully restored.

To further investigate the influence of BosR on gene expression, a *bosR* expression construct (pOY112) was created using a newly-developed *lac*-based inducible expression system [45]. In pOY112, *bosR* transcription was placed under the direct control of the IPTG-inducible PpQE30 promoter. Bb 297 transformed with pOY112 were cultivated in the presence of various amounts of IPTG. Late log-phase cells were harvested and analyzed by immunoblot. Relative to protein levels in cells grown in medium without IPTG, 50 µM of IPTG induced the production of BosR, as well as increased the levels of RpoS, OspC, and DbpA (Fig. 1C), suggesting that BosR activates expression of these genes. Gene expression in WT 297 containing the empty vector (grown under various concentrations of IPTG) was not altered (data not shown). We also complemented the *bosR* mutation in *trans* using the IPTG-inducible *bosR* expression construct pOY112. As shown in Fig. 1D, when BosR was expressed from pOY112 by IPTG, expression of RpoS, OspC and DbpA was consequently restored.

These data, consistent with previous studies [32]–[34], further corroborate that BosR activates the expression of *rpoS*, *ospC* and *dbpA*. Of note, expression of *rrp2*, *rpoN* or *bb0646* (the gene downstream from *bosR* in the *bb0648-bosR-bb0646* operon) was not affected by the *bosR* mutation (Fig. 1B), implying that the phenotypes observed were not due to impairment in the expression of *rrp2*, *rpoN*, or *bb0646*. In addition, NapA levels were found to be similar in WT, the *bosR* mutant, and the complemented strains (Fig. 1A).

### BosR controls *ospC* and *dbpA* expression through RpoS

**Figure 2:**
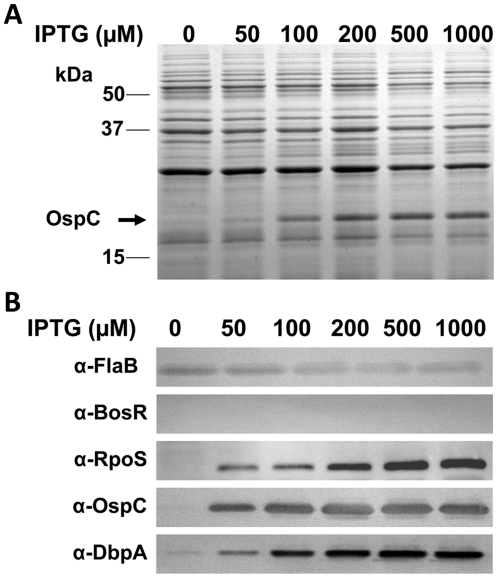
Induction of *rpoS* by IPTG results in the synthesis of OspC and DbpA in the *bosR* mutant. The *bosR* mutant OY08/A11 harboring the IPTG-inducible *rpoS* construct (pOY110) was grown at 37°C with various concentrations of IPTG and gene expression was analyzed by SDS-PAGE (A) and immunoblot (B). Approximate molecular masses are indicated at the left in kDa. The arrow in (A) indicates the position of OspC in SDS-PAGE. Specific antibodies, indicated as α-, used in the immunoblot (B) are indicated on the left.

Although our data suggested that the expression of both *ospC* and *dbpA* was activated by BosR, it remained unknown how BosR controlled the expression of these two lipoproteins. Given our finding that *rpoS* transcription was abolished in the *bosR* mutant, and the observations that expression of *ospC* and *dbpA* are directly regulated by RpoS through RpoS-specific promoters [46]–[48], we hypothesized that BosR likely regulated the expression of *rpoS* which, in turn, influences *ospC* and *dbpA*. To test this hypothesis, an IPTG-inducible *rpoS* expression construct was generated to render RpoS synthesis independent of BosR. This vector, pOY110, was then introduced into the *bosR* mutant OY08/A11. When RpoS was induced from pOY110 by IPTG, expression of OspC and DbpA was consequently rescued, although expression of BosR was absent (Fig. 2), suggesting that the controlled induction of RpoS could overcome the BosR deficiency and that BosR indirectly, rather than directly, controls OspC and DbpA expression.

### Recombinant BosR purification and metal content analysis

**Figure 3:**
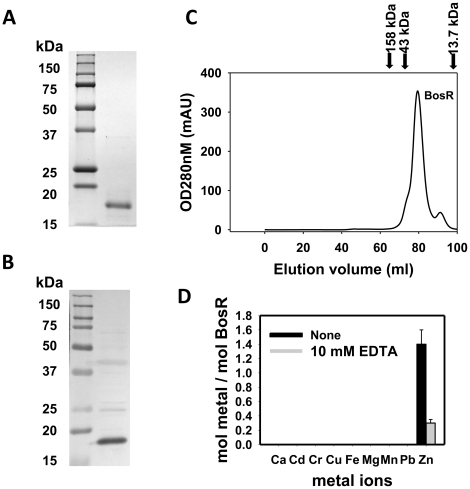
Analyses of purified recombinant BosR. (A) 12.5% (w/v) SDS–PAGE of purified recombinant BosR (right lane). Molecular masses are indicated in the left lane in kDa. (B) Native BosR in Bb cultivated in BSK-H at 37°C was probed with α-BosR. Right lane: molecular mass markers; left lane: Bb lysates. (C) Size exclusion chromatogram of purified BosR on a Superdex 200 column. Protein molecular mass standards used to calibrate the gel-filtration column are indicated by arrows (aldolase  = 158 kDa; ovalbumin  = 43 kDa; ribonuclease A  = 13.7 kDa). (D) Metal content analysis of recombinant BosR. Metal content was determined by ICP-AES.

Recombinant BosR (rBosR) was hyper-expressed in *E. coli* and purified to apparent homogeneity. SDS-PAGE analysis indicated that BosR has an apparent molecular mass of ∼18.7 kDa (Fig. 3A), which is in agreement with the apparent mass of native BosR in Bb (Fig. 3B). Furthermore, when analyzed by size-exclusion chromatography, purified BosR eluted predominantly as a dimer with a molecular mass of ∼38 kDa (Fig. 3C). Although recombinant BosR has been obtained previously and Zn^2+^ was found to affect BosR's *in vitro* binding to DNA [28]–[29], it remained unclear whether BosR contains bound metal. Therefore, metal content analysis was carried out by inductively coupled plasma atomic emission spectrometry (ICP-AES) [27], [49]. rBosR did not contain detectable levels (<0.001 ppm) of metal ions such as Fe or Mn (Fig. 3D). Rather, it contained 1.4 mol of zinc per mol of protein. Moreover, in order to remove bound metal(s) from BosR, we also dialyzed the protein in the presence of 10 mM EDTA. However, 0.3 mol of zinc/mol of proteins remained in the demetallated BosR (Fig. 3D), suggesting that the recombinant protein bound zinc avidly. Of note, these properties are typical of the dimeric bacterial Fur protein [50] or the *Bacillus subtilis* H_2_O_2_ stress response regulator PerR [51].

### BosR directly impacts *rpoS* expression

**Figure 4:**
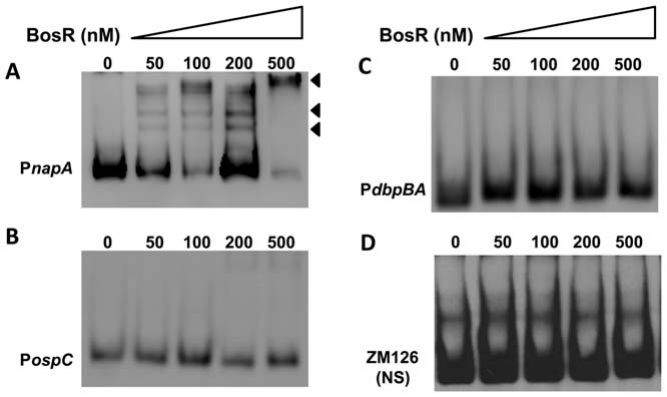
DNA-binding activity of recombinant BosR. BosR binds to the *napA* promoter (A, P*napA*), but not to the promoters of *ospC* (B, P*ospC*), *dbpBA* (C, P*dbpBA*), or probe ZM126 (D). 30 fmol of labeled DNA was incubated with various concentrations of BosR. NS, non-specific.

**Figure 5:**
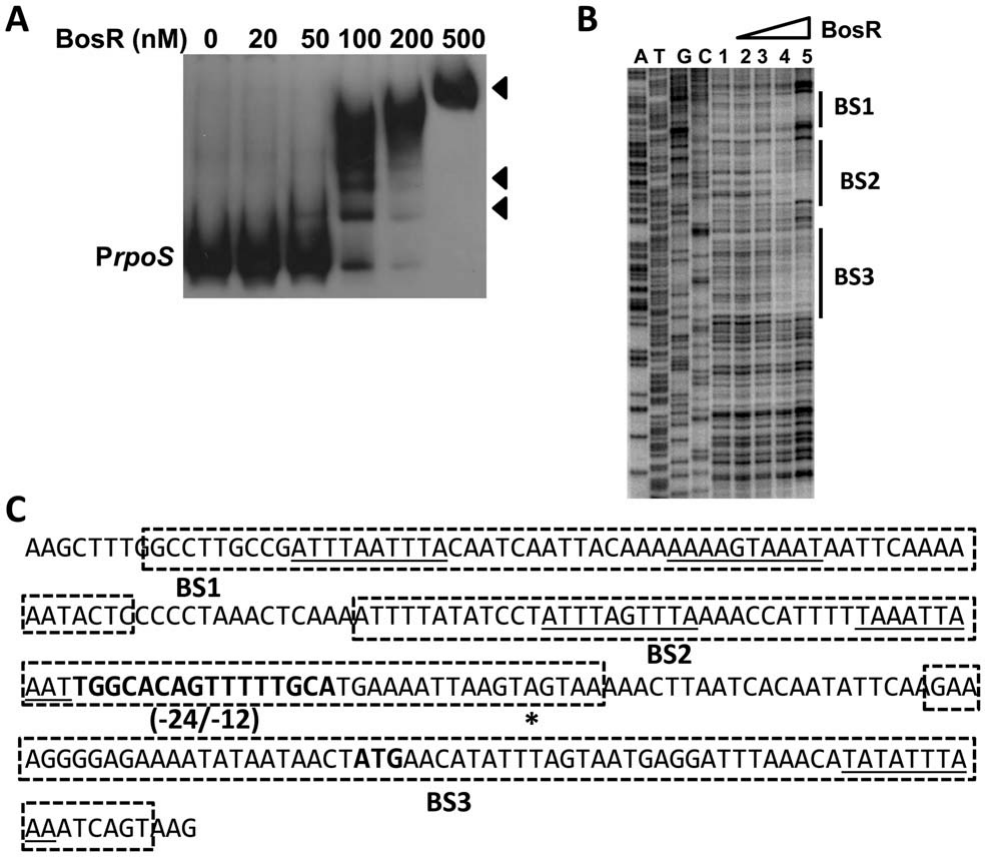
BosR binds to the *rpoS* promoter. (A) In a typical EMSA, 30 fmol of digoxigenin-labeled *rpoS* promoter (P*rpoS*) was incubated with the indicated concentrations of BosR at 37°C for 30 min. Probe name is indicated at the position of unbound DNA. Bound DNA is denoted by arrows. (B) DNase I footprinting analysis of the PrpoS probe with BosR. Lanes A, T, G and C represent sequencing ladders. Lane 1–5 contains 0, 200, 500, 1000, 1500 nM of BosR, respectively. The protected regions are marked on the right. (C) A summary of the DNase I footprinting assay results. The -24/-12 σ^54^ promoter sequence and the ATG start codon are indicated in boldface. The *rpoS* transcription start site is marked by the asterisk. The BosR protected regions (BS1-3) are indicated with the dotted-line box. The predicted direct repeat (DR) sequence is underlined.

*In silico* analysis predicted that Bb BosR contains an N-terminal winged helix-turn-helix DNA binding domain and a C-terminal dimerization domain. Three-dimensional (3D) protein modeling using the Swiss-model program (http://swissmodel.expasy.org/) indicated that the structure of the DNA-binding domain of BosR is quite similar to the *Vibrio cholerae* Fur protein [52] and the *B. subtilis* PerR protein [51] (Fig. S3), suggesting that, consistent with previous reports [28]–[29], BosR may be a DNA-binding protein. Moreover, our aforementioned data revealed that BosR impacted *rpoS* expression at the transcription level. Thus, EMSAs were performed to examine potential interactions between BosR and the *rpoS* promoter. Consistent with previous studies [28]–[29], BosR bound to the promoter of Bb *napA* (from −336 to +48, relative to the ATG start codon) (Fig. 4A). However, BosR did not bind to the *ospC* or *dbpBA* promoters under our tested conditions (Fig. 4B and C), providing support that BosR likely does not impact *ospC* and *dbpA* directly. Although BosR did not bind to the probe ZM126 that encompasses the *rpoS* promoter from −67 to −8 (Fig. 4D), BosR, in a dose-dependent manner, bound to the *rpoS* promoter (P*rpoS*) encompassing 277 bp of the *rpoS* upstream region and 245 bp of the *rpoS* encoding region (Fig. 5A). Of note, binding of rBosR generated multiple shifted bands, suggesting the possible existence of multiple BosR binding sites (BSs) in the probe. As an initial approach to identify the BosR binding sequence, DNase I footprinting assays were performed. As shown in Fig. 5B, three BosR BSs were recognized in the P*rpoS* DNA. Specifically, BosR BS1, BS2, or BS3 spanned regions of −193 to −137, −120 to −46, or −29 to +43 (relative to the ATG start codon, where A is +1), respectively (Fig. 5C).

**Figure 6:**
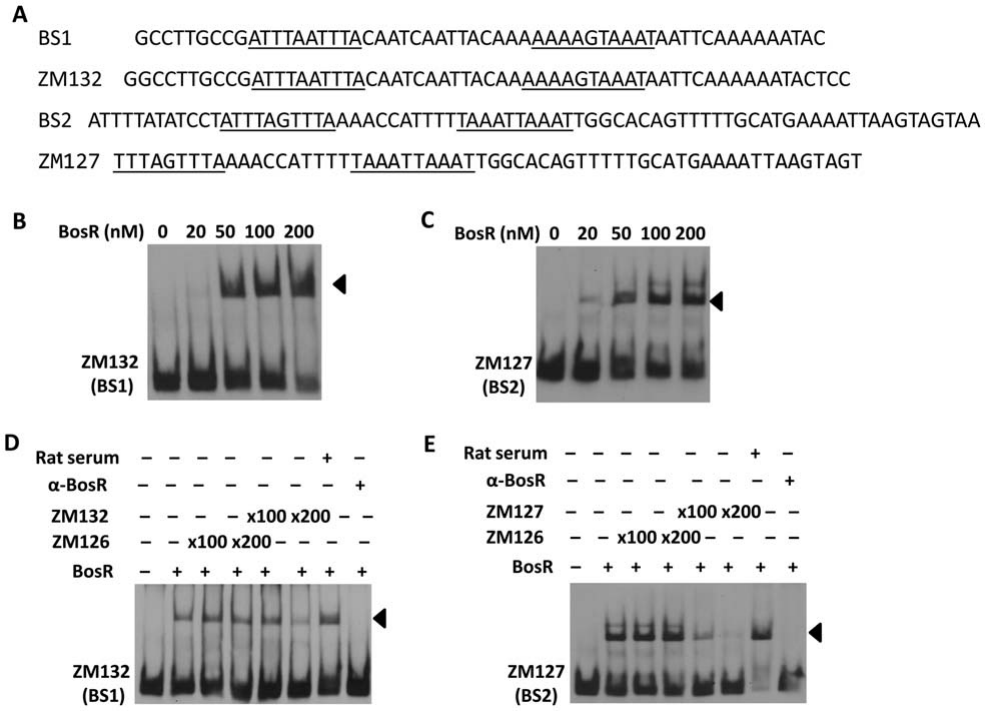
Binding of BosR to various probes containing the DNase-I protected regions. DNase-I protected regions are as indicated in Fig. 5. 30 fmol of digoxigenin-labeled probes were used in EMSAs. Probe names are indicated at the position of unbound DNA, while bound DNA is denoted by arrows. (A) Detailed sequences of probes representing BS1 and BS2. The DRs are underlined. (B, C) The concentration of BosR is designated above each lane. (D, E) 0 (−) or 50 nM (+) of BosR was used in EMSA. Inclusion (+) or exclusion (−) of competitor DNA or serum are shown as indicated. ×: fold.

**Figure 7:**
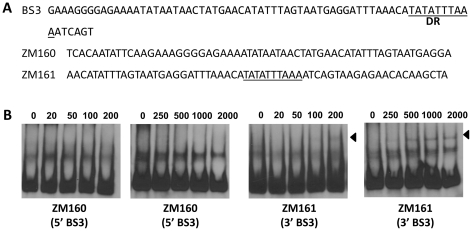
BosR binds to BS3 in P*rpoS*. (A) Probes were synthesized to represent the 5′ or 3′ sequences of BS3. The predicted DR is underlined. (B) EMSAs. The concentration of BosR (nM) is designated above each lane. Probe name is indicated below the image. Bound DNA is denoted by arrows.

To corroborate the DNase I-footprinting data, EMSA employing synthesized double-stranded (ds) DNA oligonucleotides representing different BosR BSs were performed. As shown in Fig. 6B and C, BosR bound strongly to both ZM132 (representing BS1) and ZM127 (representing BS2). Moreover, the binding of BosR to labeled BS1 (ZM132) or BS2 (ZM127) was inhibited by the addition of a 200-fold excess of unlabeled DNA, but not inhibited by the addition of a 200-fold excess of non-specific competitor ZM126 DNA (Fig. 6D and E), suggesting that BosR binds to BS1 (ZM132) or BS2 (ZM127) specifically. Binding to both probes also was abrogated by the addition of α-BosR antibody, but not by the addition of control rat serum (Fig. 6D and E), indicating that the DNA shift was indeed caused by BosR. Of note, BosR, like Fur or PerR, putatively comprises an N-terminal DNA binding motif domain and a C-terminal domain involved in protein dimerization. Both domains are essential for Fur/PerR recognizing and binding to its target DNA as a homodimer [51]–[52]. Therefore, interaction with either domain of BosR by antibody may also interrupt protein binding to DNA and prevent DNA-BosR complex formation. Similarly, we also examined the binding of BosR to BS3 using EMSA. As shown in Fig. 7, although BosR did not bind to probe ZM160 that corresponds to the 5′ sequence of BS3, the protein bound to probe ZM161 (encompassing *rpoS* from +4 to +63).

### BosR exhibits different affinities for the three binding sites

**Figure 8:**
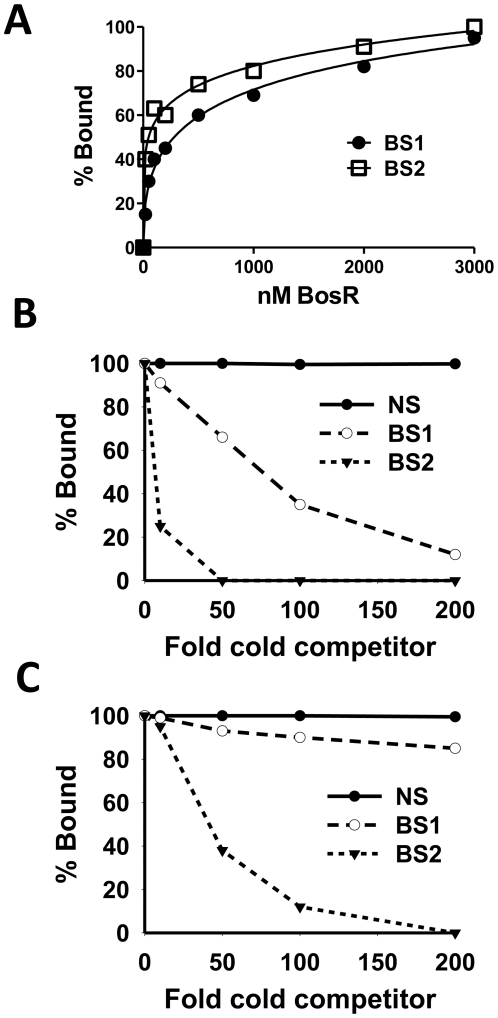
Comparison of the relative affinity of BosR for BS1 (ZM132) and BS2 (ZM127). (A) 30 fmol of labeled probe was incubated with various concentrations of BosR (nM). The membrane containing the bound and unbound DNA was detected using an enzyme immunoassay, and exposed to a Fujifilm LAS-3000 Imager (Fujifilm). Images were analyzed by using the MultiGauge V3.0 software (Fujifilm), and bands were quantified to determine the affinity of BosR for probes. (B) Competition of labeled BS1 (ZM132) with various amounts of unlabeled BS1, BS2, or non-specific (NS) DNA. (C) Competition of labeled BS2 (ZM127) with various amounts of unlabeled BS1, BS2, or NS DNA. In (B) and (C), 100 nM of BosR was used in EMSAs, and bound and unbound DNA was measured as described in (A). NS: non-specific competitor (ZM126).

EMSAs employing target DNA sequences (representing the three BosR binding sites) exposed to increasing concentrations of rBosR were used as means of inferring BosR binding affinities for the three binding sites. As shown in Fig. 6 and 7, concentrations of 50, 20, or 200 nM of rBosR induced shifts by ZM132 (BS1), ZM127 (BS2), or ZM161 (BS3), respectively, suggesting that BosR has an affinity for these DNA targets in the order of BS2>BS1>BS3. In addition, when 200 nM of rBosR was used, only a slight proportion (<10%) of ZM161 (BS3) was shifted (Fig. 7), and probe ZM161 could not be saturated even by 10,000 nM of BosR (data not shown). To more precisely assess the affinity of BosR for BS1 and BS2, we measured the amount of bound DNA as a function of BosR concentration in EMSA assays (Fig. 8A). The dissociation binding constants (*Kd*) for BS1 (ZM132) or BS2 (ZM127) were 210.2 or 36.6 nM, respectively. The relative affinities of these two DNA elements for BosR were also assessed by competition EMSA analysis (Fig. 8, B and C). Binding of labeled BS1 or BS2 was not inhibited by the non-specific competitor ZM126 (NS), but was inhibited by unlabeled (cold competitor) BS1 or BS2, respectively. Moreover, binding of labeled BS1 was inhibited approximately 90% by the addition of 200-fold unlabeled BS1, but was completely competed out by 50-fold unlabeled BS2 (Fig. 8B). The addition of 200-fold of unlabeled BS1 competed out only 15% of BS2 binding (Fig. 8C). These data indicate that BosR has a higher affinity for BS2 than for BS1.

### Identification of a novel direct repeat sequence critical for BosR binding

*In silico* analysis indicated that BosR contained two potential CX_2_C Zn^2+^ binding motifs in its C-terminus (located at residues of 114–117 and 153–156). These types of Zn^2+^ structural sites are crucial for Fur dimerization and binding DNA as a homodimer [30], [53]. Accordingly, we found that BosR bound Zn tightly (Fig. 3D). Moreover, consistent with previous observations [28], our purified BosR appeared to exist principally as a dimer in solution (Fig. 3). Therefore, BosR may bind to the *rpoS* promoter as a homodimer, suggesting that the BosR binding sequence(s) may be a direct repeat (DR) sequence. In agreement with this assumption, close inspection of the three BosR BSs revealed a DR sequence (TAAATTAAAT) (Fig. 5C). Of note, this sequence also consists of two contiguous pentamer direct repeats (TAAAT). More specifically, BS1 contains one perfect DR at its 5′ sequence and one imperfect DR at its 3′ sequence; BS2 contains one perfect DR and one imperfect DR in the sequence upstream of the -24/-12 RpoN binding site; and BS3 contains one imperfect DR sequence at its 3′. Of note, in both BS1 and BS2, the DR1 and DR2 are located on opposite DNA strands.

**Figure 9:**
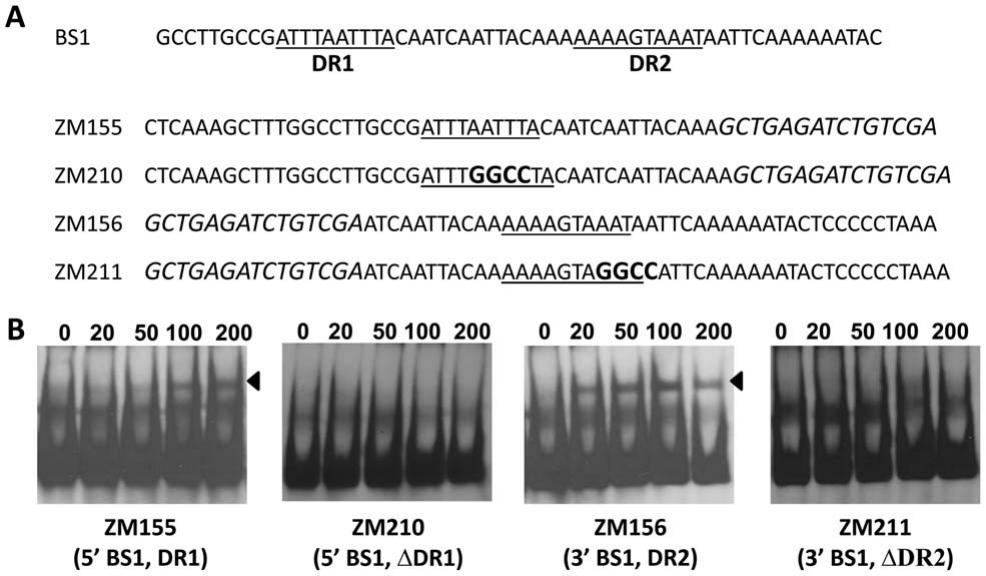
The DRs in BS1 are essential for BosR binding. (A) Detailed sequences of probes representing BS1. The DRs are underlined. Scrambled sequences are italicized. Mutated nucleotides are indicated in boldface. (B) EMSAs. The concentration of BosR (nM) is designated above each lane. Probe name is indicated below the image. Bound DNA is denoted by arrows.

We hypothesized that if the DR is essential for binding to BosR, mutational changes in the sequence should abolish or inhibit BosR binding. Along these lines, we initially synthesized two DNA fragments, ZM155 and ZM156, representing the 5′ or 3′ of BS1, respectively (Fig. 9A). Both ZM155 and ZM156 contain one DR sequence. After labeling these DNA fragments with digoxigenin, each DNA fragment was mixed with BosR and EMSAs were performed. As shown in Fig. 9B, BosR bound to both fragments. However, when the DR was mutated, the binding of BosR to either DNA fragment was abolished (Fig. 9B), strongly supporting the notion that the DR sequence is critical for BosR binding.

**Figure 10:**
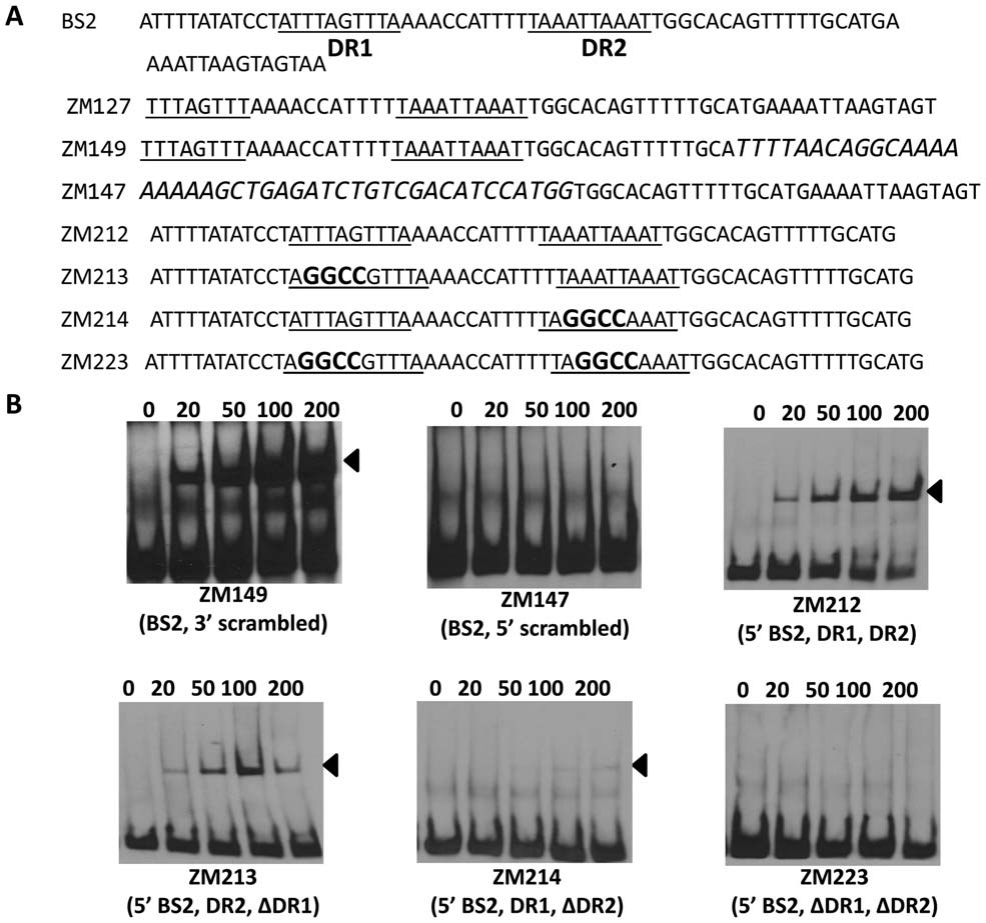
Analysis of BosR binding to BS2. (A) Detailed sequences of probes representing BS2. The DRs are underlined. Scrambled sequences are italicized. Mutated nucleotides are indicated in boldface. (B) EMSAs. The concentration of BosR (nM) is designated above each lane. Probe name is indicated below the image. Bound DNA is denoted by arrows.

Using this same strategy, we also examined the two DRs in BS2. As shown in Fig. 10, although BosR still bound to probe ZM149 having sequences downstream of the -24/-12 site scrambled, BosR binding to BS2 was abolished when sequences upstream of the -24/-12 site were scrambled (ZM147), suggesting that the functional BosR binding sites are located in the sequence upstream of the -24/-12 site. Because sequences flanking the binding motif often play important roles in protein-DNA interactions, we synthesized another dsDNA (ZM212) to represent the 5′ of BS2, allowing added flanking sequences to the predicted DR sequences. EMSAs indicated that BosR still bound to probe (ZM213) with the DR1 mutated (Fig. 10B). When a mutation was introduced into DR2 (ZM214), BosR binding was dramatically reduced (Fig. 10B). Moreover, when both DR sequences were mutated, protein binding was completely abolished (Fig. 10B). These data suggest that DR1 and DR2 in BS2 are required for BosR binding.

### Genome-wide distribution of the DR sequence essential for BosR binding

To identify other Bb genes potentially regulated by BosR, Katona et al. [28] performed a BlastN analysis based on the Per box consensus sequence. However, the DR identified in our current study is disparate from the Per box. To uncover additional BosR-regulated genes, we searched the Bb genome by using the Regulatory Sequence Analysis Tools (http://rsat.ulb.ac.be/rsat), and queried for genes containing a perfect DR sequence in putative promoter regions. Gene promoter regions were defined as sequences from −400 to +50 bp (relative to the ATG start codon). The results are shown in Table 2. A total of 60 Bb genes were found to harbor one or multiple perfect DR sequence in their promoter regions. Thirty-one genes were located on the main chromosome, and 29 genes were on linear or circular plasmids. Of the 31 chromosomal genes, 16 genes encode proteins with assigned functions and15 genes encode hypothetical proteins. More importantly, 13 of these genes were found to be regulated by BosR in our recent microarray analysis [34], further supporting that the DR sequence is important for BosR binding.

**Table 2:** *Borrelia burgdorferi* genes containing a perfect direct repeat sequence (TAAATTAAAT) in their putative promoter regions.

ID	Gene	Function	matching sequence
BB0007		hypothetical protein	ttatTAAATTAAATtgtt
BB0008		conserved hypothetical protein	ttatTAAATTAAATtgtt
BB0020	*pfpB*	Pyrophosphate—fructose 6-phosphate 1-phosphotransferase, beta subunit	atcaTAAATTAAATatta
BB0036	*parE*	DNA topoisomerase IV	tttaTAAATTAAATttta
BB0055	*tpiA*	triosephosphate isomerase	cagaTAAATTAAATttat
BB0086		conserved hypothetical protein	gtttTAAATTAAATttcc
BB0097		hypothetical protein	cactTAAATTAAATctca
BB0141	*mtrC*	membrane fusion protein	tttcTAAATTAAATgata
BB0141			aagaTAAATTAAATtatg
BB0259		hypothetical protein	atttTAAATTAAATtcaa
BB0300	*ftsA*	cell division protein	atcaTAAATTAAATgctt
BB0306		conserved hypothetical protein	ggttTAAATTAAATgaat
BB0322		hypothetical protein	ttctTAAATTAAATtata
BB0323		hypothetical protein	ttctTAAATTAAATtata
BB0337	*eno*	enolase	gtcaTAAATTAAATaatc
BB0524		inositol monophosphatase	ttgaTAAATTAAATattt
BB0564		hypothetical protein	aggtTAAATTAAATttaa
BB0564			ttggTAAATTAAATttta
BB0565	*cheW-2*	purine-binding chemotaxis protein	ttggTAAATTAAATttta
BB0565			aggtTAAATTAAATttaa
BB0578	*mcp-1*	methyl-accepting chemotaxis protein	gtttTAAATTAAATtaaa
BB0578			aaatTAAATTAAATtaaa
BB0578			atttTAAATTAAATttac
BB0580		conserved hypothetical integral membrane protein	ataaTAAATTAAATgacc
BB0592		hypothetical protein	aaaaTAAATTAAATtgag
BB0601	*glyA*	serine hydroxymethyltransferase	caatTAAATTAAATattt
BB0608	*pepD*	aminoacyl-histidine dipeptidase	attaTAAATTAAATccaa
BB0608			tgatTAAATTAAATccat
BB0668	*flaA*	flagellar filament outer layer protein	ttgaTAAATTAAATttta
BB0672	*cheY-3*	chemotaxis response regulator	cttcTAAATTAAATtttg
BB0739		hypothetical protein	aaatTAAATTAAATattt
BB0771	*rpoS*	RNA polymerase sigma factor	ttttTAAATTAAATtggc
BB0771			attgTAAATTAAATcggc
BB0791	*tdk*	thymidine kinase	atacTAAATTAAATaac
BB0798		putative competence protein F	atatTAAATTAAATgagt
BB0846		hypothetical protein	aaaaTAAATTAAATataa
BB0849		hypothetical protein	aaaaTAAATTAAATataa
BB0851		putative exported protein	atctTAAATTAAATtgat
BBA05		antigen, S1	ttcaTAAATTAAATtacc
BBA33		hypothetical protein	gaaaTAAATTAAATtttc
BBB03		hypothetical protein	atatTAAATTAAATtata
BBB29	*malX*	PTS system, maltose and glucose-specific IIABC component	atttTAAATTAAATttag
BBC06	*eppA*	exported protein A	tttcTAAATTAAATattt
BBF01		erpD protein, putative	ctccTAAATTAAATaaaa
BBF22		protein p23, putative	tttaTAAATTAAATaaaa
BBF24		plasmid partition protein, putative	gaaaTAAATTAAATtcac
BBG02		conserved hypothetical protein	aaagTAAATTAAATaaca
BBG08		plasmid partition protein, putative	taaaTAAATTAAATtctt
BBI16		hypothetical protein	taatTAAATTAAATattt
BBI16			taatTAAATTAAATaaat
BBI36		antigen, P35, putative	acttTAAATTAAATacta
BBI37		hypothetical protein	acttTAAATTAAATacta
BBI38		hypothetical protein	acttTAAATTAAATacta
BBI39		hypothetical protein	acttTAAATTAAATatta
BBJ32		hypothetical protein	ttttTAAATTAAATgaat
BBJ39		hypothetical protein	aaccTAAATTAAATatta
BBJ40		hypothetical protein	aaccTAAATTAAATatta
BBJ41		antigen, P35, putative	acctTAAATTAAATatta
BBK18		conserved hypothetical protein	tccaTAAATTAAATcaat
BBL04		hypothetical protein	gagaTAAATTAAATttta
BBM09		conserved hypothetical protein	aaagTAAATTAAATccga
BBN41		hypothetical protein	gaacTAAATTAAATgaag
BBO04		hypothetical protein	gagaTAAATTAAATttta
BBO09		conserved hypothetical protein	aaagTAAATTAAATccga
BBO33		conserved hypothetical protein	tattTAAATTAAATagat
BBP04		hypothetical protein	gagaTAAATTAAATttta
BBQ72		hypothetical protein	taatTAAATTAAATccat
BBS04		hypothetical protein	gagaTAAATTAAATttta

1, Candidate genes were identified by searching *B. burgdorferi* B31 genome using “Regulatory Sequence Analysis Tools”. Gene promoter was defined as the region encompassing sequences from -400 to +50 (relative to a putative translation start ATG).

2, Four flanking nucleotides are also shown.

**Figure 11:**
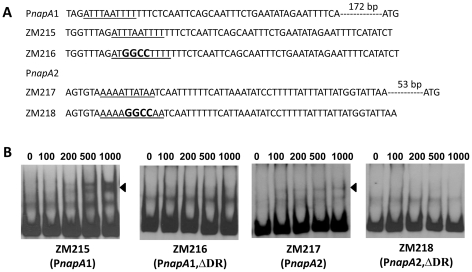
The DRs in P*napA* are required for BosR binding. (A) Detailed sequences of the probes used in EMSAs. P*napA*1 represents the BosR-protected region identified by Boylan, *et al.* [29]. P*napA*2 represents the probe used by Katona, *et al.* [28]. The DRs are underlined. Mutated nucleotides are indicated in boldface. (B) EMSAs. The concentration of BosR (nM) is designated above each lane. Probe name is indicated below the image. Bound DNA is denoted by arrows. The numbers in parenthesis indicate numbers of omitted nucleotides (dash lines) between the probe and the ATG start codon.

**Figure 12:**
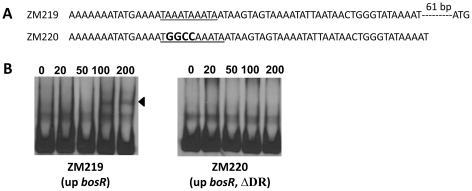
Analysis of the DR in the upstream region of *bosR*. (A) Sequences of the probes used in EMSAs. The DR is underlined. Mutated nucleotides are indicated in boldface. (B) EMSAs. The concentration of BosR (nM) is designated above each lane. Probe name is indicated below the image. Bound DNA is denoted by arrows. The numbers in parenthesis indicate numbers of omitted nucleotides (dash lines) between the probe and the ATG start codon.

Previously, BosR was reported to bind to the Bb *napA* promoter (P*napA*). Using footprinting assays, Boylan *et al.* [29] found that BosR protected a 50-bp region located at -222 to -173 (relative to the ATG start codon) in P*napA*. Katona *et al.* [28], using EMSAs, reported that BosR also bound to a DNA fragment encompassing P*napA* from −152 to +3. In addition, the latter researchers also reported that BosR bound to upstream regions of *bosR*. Interestingly, these two genes were not identified in our search (Table 2). However, when scrutinizing the upstream regions of *bosR* and *napA*, multiple imperfect DR sequences were detected (Fig. 11A, 12A). Therefore, EMSAs using synthesized dsDNA were employed to examine the roles of these imperfect DR sequences in BosR binding. Specifically, two dsDNA fragments, ZM215 and ZM217, were used to represent the BosR binding region in P*napA* identified in previous studies [28]–[29]. As shown in Fig. 11B, BosR bound to both DNA fragments. When a mutation was introduced into the DR, binding of BosR to each probe was abolished. Similar data were also obtained for the probe ZM219 representing the *bosR* upstream region; BosR binding to the probe was abolished when the predicted DR was mutated (ZM220) (Fig. 12B). These data further substantiate the critical role of the DR in BosR binding.

### BosR and RpoN bind to distinct sites in the *rpoS* promoter regions

**Figure 13:**
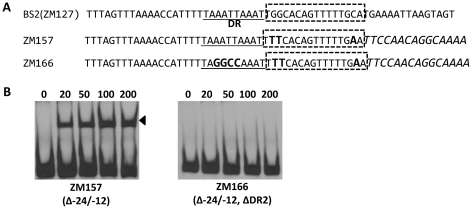
Different key nucleotides are required for BosR or RpoN binding to the *rpoS* promoter. (A) Sequences of the probes used in EMSAs. The DR is underlined. The -24/-12 region is indicated with the dotted-line box. Scrambled sequences are italicized. Mutated nucleotides are indicated in boldface. (B) EMSAs. The concentration of BosR (nM) is designated above each lane. Probe name is indicated below the image. Bound DNA is denoted by arrows.

Bb RpoN activates *rpoS* directly through a canonical -24/-12 promoter, and mutation of G at -24 to T in the *rpoS* promoter resulted in a significant diminishment of *in vitro* RpoN binding and a dramatic decrease in *rpoS* expression [8], [11], [24]. Our analysis of BS2, the site exhibiting the highest affinity for BosR among the three BosR binding sites in the *rpoS* promoter, revealed that a perfect DR sequence is located just upstream and adjacent to the -24/-12 σ^54^ promoter (Fig. 13A). Given the importance of this locus for *rpoS* transcription, we further examined this site more closely using EMSA. As shown in Fig. 13B, when nucleotides in the -24/-12 site were mutated (ZM157, mutations of G-24T, G-25T, and C-12A), the binding of BosR was not altered. In contrast, when a mutation was introduced into the DR upstream of the -24/-12 site (ZM166, AATT replaced by GGCC), BosR binding was abolished (Fig. 13). These data strongly suggest that the key nucleotides for the binding of BosR and σ^54^ to the *rpoS* promoter are different.

## Discussion

The essential role of the RpoN-RpoS pathway for virulence expression by the Lyme disease bacterium is now well documented [8]–[11], [13], [16]–[17], [24]–[25]. However, a number of the molecular details involved in the activation of this pathway have remained obscure. Whether Rrp2, as an EBP, is present as a dimer and assembles into a functional hexamer or heptamer when activated is unknown. It is also unclear how Rrp2 interacts with Eσ^54^ to modulate RpoN-dependent *rpoS* expression in the absence of demonstrable specific binding to the *rpoS* promoter or upstream region [8], [24], but there is precedence for this anomaly in another bacterial pathogen, *Campylobacter jejuni* (its EBP FlgR activates the σ^54^-dependent flagellar genes independent of DNA-binding) [54]. Moreover, the mammalian host factors that influence *rpoS* expression [12]–[13] have not been identified. Recently it was shown in Bb strain B31 that a mutation in *bosR* led to a loss of mouse infectivity, as well as a block in the expression of the virulence-associated factors OspC and DbpA [32], [34]–[35]. Given that both *ospC* and *dbpA* are under the control of RpoS [46]–[48], we proposed that BosR somehow was involved in the activation of the RpoN-RpoS regulatory pathway [34]. However, these data and the resultant hypothesis emanated from studies involving only one virulent strain (strain B31) of Bb. It has long been established that genetic regulators and control mechanisms can vary widely among strains of the same virulent species of pathogenic bacteria [36]–[38]. Hence, the question remained whether the novel RpoN-RpoS regulatory pathway and the important role of BosR were common to other virulent strains of Bb. The results of our study herein now confirm that the inactivation of *bosR*, which prevents activation of the RpoN-RpoS pathway by blocking the expression of *rpoS*, and, in turn, prevents expression of the virulence-associated genes *ospC* and *dbpA*, is not unique to a single virulent strain of Bb. BosR and its control over RpoN-RpoS activation thus appears to be an important global regulatory pathway essential for virulence expression by the Lyme disease spirochete.

Our findings also provide new insights into the function of BosR. BB0647 (BosR) was originally predicted to be a Fur homologue [7], [28]. Given the lack of precedence for interplay between a Fur homologue and an alternative sigma factor, such as RpoN or RpoS, in other bacteria, it was entirely unexpected that BosR would play a role in the induction of the RpoN-RpoS pathway [32], [34]. Furthermore, the nomenclature of “BosR”, as *Borrelia* oxidative stress regulator, was derived from the sequence similarity of BosR to the *B. subtilis* PerR, and an observation [29] that, in the heterologous *E. coli*, BosR activated the expression of Bb *napA* implicated in the oxidative stress response [55]. It was previously reported that NapA production was inhibited in a *bosR* mutant of Bb, and that the mutant displayed an *in vitro* growth defect [32]. Our former B31 mutant deficient in *bosR* exhibited no such growth defect, and the expression of *napA* was not significantly altered in the *bosR* mutant [34]; these same wild-type-like phenotypes were also observed in our current *bosR* mutant derived from strain 297 (Fig. 1B, Fig. S2B). Given these more recent findings, and the current paucity of compelling data that directly link BosR to an oxidative stress response in Bb, it is thus still premature to conclude that BosR is involved in modulating oxidative stress in Bb. Finally, despite the characterization of BosR as a Fur homologue, it also remains unclear whether BosR plays a role in regulating transition metal homeostasis in Bb.

Our EMSA data clearly indicated that BosR binds to the *rpoS* promoter, suggesting that BosR directly influences *rpoS* expression. Moreover, DNA footprinting assays revealed three BosR-protected regions in *rpoS*. The occupation of multiple, rather than one, binding sites might stabilize and secure BosR binding to the *rpoS* promoter region. BosR exhibited binding affinity for these three sites in the order of BS2>BS1>BS3. Among these three sites, BS2 was found juxtaposed to and partially overlapping with the -24/-12 RpoN binding site, whereas BS1 is located upstream of BS2. BS3 is located in sequences downstream of BS2, or more specifically, in the RpoS-encoding region. A previous study revealed that, when Bb was grown *in vitro*, a minimal P*rpoS* (starting from the -24/-12 RpoN binding site), which contains only one intact BosR BS (BS3), is able to express RpoS at the same level as P*rpoS* containing all three BosR BSs [24]. Furthermore, RpoS expressed from the minimal P*rpoS* restored mouse infectivity to the *rpoS* mutant [24]. These data imply that P*rpoS* with only BS3 is sufficient to drive *rpoS* transcription, suggesting that our data of BosR binding to BS3 may be physiologically relevant. However, BS1 and BS2 may be required to coordinate *rpoS* expression under different *in vivo* conditions of the two diverse niches of Bb's complex life cycle. Our unanticipated finding that BS3 for BosR is located within the RpoS-encoding region is rare but not unprecedented for transcriptional activators; binding sites for other bacterial regulatory proteins have been noted to occur in the coding regions of their target genes [56]–[57]. It is thus possible that BS3 located in the *rpoS* encoding region may somehow strengthen the binding of BosR to P*rpoS*, and then cooperate in opening the RpoN-RNAP closed complex. Or, BosR binding at BS3 might allow *rpoS* expression to be controlled more tightly, especially if *rpoS* transcription requires transient modulation.

A major finding of this study is the identification of a novel DNA binding sequence for BosR. As a Fur homologue, BosR dimers were reported [28]–[29] to bind *in vitro* to the Bb *napA* promoter, the upstream regions of *bosR* and *bb0646*, and DNA containing a Fur box (GATAATGATAATCATTATC) or Per box (TTATAAT-ATTATAA). In general, the Fur box is interpreted as two 9-bp inverted repeats (GATAATGAT), or two heptamer inverted repeats (TGATAAT), or three hexamer repeats (GATAAT), whereas the Per box is recognized as two inverted repeat (TTATAAT) [53], [58]. Despite the facts that the *rpoS* promoter contains neither a Fur (or Per) box, nor has significant similarity with the Bb *napA* promoter or the upstream sequences of *bosR* and *bb0646*, BosR binds to the *rpoS* promoter. More importantly, we identified a DR sequence (TAAATTAAAT) that is critical for BosR binding. This assertion is strongly supported by several lines of evidence. First, the DR sequence is present in all three BosR BSs. Second, the DR sequence was identified in the promoter regions of 13 genes already known to be influenced by BosR. Third, imperfect DR sequences are present in previously-established BosR-binding DNA fragments, such as P*napA* and a *bosR* upstream region. Finally, mutations in the DR severely reduced or completely abolished DNA binding by BosR. Of note, the DR sequence is markedly different from the direct or inverted repeats present in Fur or Per boxes. Thus, BosR appears to be able to recognize different DNA sequences, including the Fur box consensus, the Per box consensus, and the *rpoS* promoter element (containing the DR sequence). Such promiscuous DNA recognition activity has been observed previously for the *Bradyrhizobium japonicum* Fur protein [59]; in addition to binding to the Fur box consensus, *B. japonicum* Fur also binds *in vitro* to the *irr* promoter (with similar affinity), but, the *irr* promoter does not contain a Fur box. Rather, it contains three essential direct repeat sequences of TGCATC that differ markedly from the direct repeats (GATAAT) or inverted repeats (GATAATGAT) in the Fur box [59]. The mechanistic details of this anomaly remain unknown. One possibility for BosR is that its binding properties *in vitro* may depend largely on DNA conformation. However, when analyzing the conformation of the dsDNA (including DNA containing mutated DRs) used in our EMSAs by PREDICTOR (http://www.farwer.staff.shef.ac.uk/PREDICTOR), which is a program calculating the three-dimensional atomic structure of dsDNA, no obvious differences in DNA conformation were revealed. Moreover, although BosR is predicted to share a similar three dimensional structure with Fur and the PerR protein (Fig. S3), BosR may harbor some subtle, unique structural feature(s) (undetected by protein modeling) that confer its DNA binding traits. Alternatively, under different *in vivo* conditions (tick vector or mammalian hosts), BosR may display alternative structural conformations that differentially regulate gene expression. As such, differing conformations may prompt BosR to bind to different DNA sequences (or with varying affinities). It is perhaps noteworthy that, after decades of intensive work, there is still much controversy over the molecular mechanisms and biochemistry of how Fur operates as a transcriptional repressor [60]. Thus, it is not surprising that there is yet much to learn regarding the molecular mechanism(s) that allow BosR to act as a regulator. Nonetheless, our finding that BosR, as a Fur or PerR homolog, recognizes disparate DNA sequences not only hints that the well-established model for Fur (or PerR)-DNA interaction may warrant further refinements, but also suggests that BosR employs mechanisms different from Fur or PerR to regulate gene expression. In addition, the recognition that BosR depends on a novel direct repeat for its binding to the *rpoS* promoter serves as a strong foundation for further mechanistic studies.

Several aspects of our study engender a number of provocative possibilities surrounding the function of BosR as a transcriptional enhancer for *rpoS*. First, our mutagenesis experiments revealed that different key nucleotides are required for BosR or RpoN binding to the *rpoS* promoter, implying that BosR and RpoN may bind to different faces of the DNA helix (comprising the *rpoS* promoter). Second, that BS2 is immediately adjacent to the -24/-12 RpoN binding site tempts speculation that BosR and RpoN (and possibly Rrp2) may interact with one another at the -24/-12 site to initiate *rpoS* transcription. However, results from *E. coli*-based two-hybrid assays thus far have failed to show interactions between BosR and RpoN (or Rrp2) (unpublished data). These results, however, are not unexpected, because there is no precedence for interactions between Fur/PerR proteins and RpoN or an EBP (e.g. Rrp2) in any bacterial system. Despite that, it is not impossible that BosR may transiently interact with RpoN or Rrp2 *in vivo*. It also remains possible that BosR may act as a critical molecule to recruit Rrp2 and/or RpoN to the *rpoS* promoter and the Eσ^54^-CC. The binding of BosR dimers to one face of the *rpoS* promoter at multiple sites may lead to DNA bending or other conformational changes that may facilitate the binding of Eσ^54^ to the -24/-12 site on the other strand of the promoter.

BosR, Fur, and PerR share structural similarity (Fig. S3) and all three are zinc- (or other metal-) containing regulatory proteins. However, there are other key features of BosR that markedly distinguish it from its putative Fur or PerR homologs. With Fur, metal-dependent DNA binding acts primarily as a repressor to avoid cellular iron toxicity [30], [53], [58], [60]. Its positive regulatory role is often indirect, via the Fur-regulated anti-sense regulatory small RNA, RhyB [30], [60]. In the case of PerR, transcriptional activation of its target genes (involved in protecting the bacterial cell against oxidative stress) occurs when the metal-bound PerR dissociates from the promoter [51], [60]. Of the three regulators, BosR is the only one that works in concert with an alternative sigma factor (RpoN) and an EBP (Rrp2), and the only one that appears to activate gene transcription directly by DNA binding. However, at this time we cannot rule out the less likely possibility that BosR may prevent the binding of a repressor that blocks *rpoS* transcription. Nonetheless, from the metal (zinc) content of rBosR, it is tempting to speculate that BosR's DNA-binding activity may be metal-dependent. Although zinc was found in rBosR, the question remains whether zinc is the physiologically relevant metal that confers normal activity to native BosR. It is thus not out of the realm of possibility that other metal(s) may be physiological relevant during Bb's existence in ticks or mammalian hosts. Further studies are warranted to investigate these possibilities, although many of the potential experimental approaches have substantial obstacles.

Our findings also reveal a new aspect of bacterial σ^54^-dependent gene activation and expands our understanding of transcriptional regulation by alternative sigma factors in general. Traditionally, for all known bacterial σ^54^-dependent genes, transcriptional activation requires only the cognate activator EBP (ATPase) [3]–[4]. For some σ^54^-dependent promoters, maximal induction relies on one or several auxiliary factors. For example, IHF, as a DNA-bending protein, can promote the interaction between Eσ^54^ and an EBP via DNA looping. In this case, however, IHF acts only as an architectural element to facilitate formation of the loop. It is the EBP, rather than the IHF protein, that modulates σ^54^-dependent gene expression [61]. In *E. coli* and *Salmonella typhimurium*, in the presence of arginine, the arginine repressor ArgR can induce the expression of the σ^54^-dependent *astCADBE* operon [62]–[63]. Nonetheless, ArgR plays only an accessory, rather than essential, role in the expression of *astCADBE*. In the absence of ArgR, genes are still expressed, although at more moderate levels. In response to flavonoids, the *Azorhizobium caulinodans* transcriptional activator NodD induces the transcription of NifA-RpoN-controlled NodA operon at an early stage [64]. In mature nitrogen-fixing nodules, the *nodA* gene is still transcribed in the *nodD* mutant in response to nitrogen-oxygen availability. In addition to the putative EBP (Rrp2), BosR is directly involved in the transcriptional activation of σ^54^-dependent *rpoS* in Bb. Unlike ArgR, NodD, and other accessory factors involved in maximizing the induction of σ^54^-dependent genes, BosR is essential for *rpoS* transcription in Bb. To our knowledge, this is the only demonstration, in any bacterial σ^54^-dependent transcriptional system, that transcription of a σ^54^-dependent gene requires an additional transcriptional activator. Bioinformatics indicate that homologues of BosR and σ^54^ are not only conserved in other *Borrelia* species (such as *B. garinii* and *B. afzelii*), but may be encoded in numerous other bacterial species including *Bordetella*, *Burkholderia*, *Shewanella*, *Campylobacter*, *Clostridium*, *Bacillus*, *Listeria*, and others. Given this wide distribution of BosR homologs, our study may have broader significance in understanding the regulatory control over RpoN- or RpoS-dependent genes in other pathogenic bacteria.

## Materials and Methods

### Strains and culture conditions

Strains and plasmid used in this study are listed in Table 3. Infectious Bb strain 297 [65] was used as the WT strain throughout this study. Bb was routinely cultured at 37°C and 5% CO_2_ in either BSK-II medium [66] or BSK-H medium (Sigma) supplemented with 6% rabbit serum (Pel-Freeze). When appropriate, supplements were added to media at following concentrations: kanamycin, 160 µg/ml; streptomycin, 150 µg/ml. Spirochetes were enumerated by dark-field microscopy.

**Table 3:** Strains and plasmids used in this study.

Strain or plasmid	Description	Source
Strains		
*B. burgdorferi*		
297	infectious, low-passage *B. burgdorferi*	[65]
OY08/A11	Bb 297, Δ*bosR*::Kan	This study
OY08/F4	Bb 297, Δ*bosR*::Kan	This study
OY33/A6	OY08/A11 transformed with pOY83, complemented strain	This study
OY33/F7	OY08/F4 transformed with pOY83, complemented strain	This study
OY57	OY08/A11 transformed with pOY110	This study
OY62	OY08/A11 transformed with pOY112	This study
*E. coli*		
TOP10	F*^−^ mcrA Δ(mrr-hsdRMS-mcrBC) f80lacZΔM15 ΔlacX74 recA1 araD139 Δ(ara-leu)7697 galU galK rpsL (Str^R^) endA1 nupG*	Invitrogen
BL21 (DE3)	F*– ompT hsdSB(rB–, mB–) gal dcm* (DE3)	Invitrogen
reporter strain	*Δ(mcrA)183 Δ(mcrCB-hsdSMR-mrr)173 endA1 supE44 thi-1 recA1 gyrA96 relA1 lac [F' laqI^q^ bla lacZ Kan^r^]*	Agilent
Plasmids		
pJD54	*B. burgdorferi*/*E. coli* shuttle vector with P*flgB-aadA* [68]	
pBT	cloning vector	Agilent
pTRG	cloning vector	Agilent
pBT-LGF2	λcI-Gal4 fusion	Agilent
pTRG-Gal11p	αRNAP-Gal11 fusion	Agilent
pJSB252	pJD7::P*pQE30*-Bb*luc*+ and P*flaB*-Bb*lacI* (divergent);	[45]
pET SUMO	protein expression vector	Invitrogen
pOY24	plasmid used to create *bosR* mutation	[34]
pOY63	promoterless *luc*_Bb+_ from pJD48 cloned into pJD54	[47]
pOY73	*bosR* cloned into pET SUMO	This study
pOY83	plasmid used to complement *bosR* mutation	[34]
pOY99.2	the P*flaB*-Bb*lacI*-PpQE30 cassette from pJSB252 cloned into pJD54	This study
pOY110	Bb 297 *rpoS* cloned into pOY99.2	This study
pOY112	Bb 297 *bosR* cloned into pOY99.2	This study
pOY135	Bb *bosR* cloned into pBT	This study
pOY136	Bb *rrp2* cloned into pBT	This study
pOY137	Bb *bosR* cloned into pTRG	This study
pOY138	Bb *rrp2* cloned into pTRG	This study
pOY139	Bb *rpoN* cloned into pTRG	This study

### Ethics statement

This study was carried out in strict accordance with the recommendations in the *Guide for the Care and Use of Laboratory Animals* of the National Institutes of Health. All animal procedures were approved by Institutional Animal Care and Use Committee at UT Southwestern Medical Center (Animal Protocol Number 0165-07-14-1).

### Construction of *bosR* mutants and complement

The *bosR* mutant OY08 was created by allelic exchange in Bb 297 using a suicide vector pOY24 [34]. The mutation in *bosR* was *cis*-complemented by transforming a suicide vector, pOY83 [34], into the *bosR* mutant, generating OY33. Transformation of Bb was performed as previously described [67]. Plasmid contents of all Bb strains were determined by PCR using specific primers.

### Generation of *lac*-inducible gene expression constructs

To artificially control BosR or RpoS expression in Bb, gene expression constructs were generated using a newly-developed *lac*-based inducible expression system [45]. First the P*flaB*-Bb*lacI*-PpQE30 cassette from pJSB252 [45] was ligated into pJD54 digested with BglII and BamHI, which generated pOY99.2. Then *rpoS* or *bosR* was amplified from Bb 297 and cloned into pOY99.2 digested with NdeI and BglII, generating pOY110 or pOY112, respectively. In these constructs, *rpoS* or *bosR* transcription was directly controlled by the IPTG-inducible T5 promoter in pQE30 (PpQE30).

### *Bb* infection of mice

The infectivity of Bb clones was assessed using the murine needle-challenge model of Lyme borreliosis [43]–[44]. C3H/HeN mice (Charles River Laboratories) were infected via intradermal injection with various concentrations of Bb. At 4 weeks post inoculation, mice were sacrificed and skin, heart, and joint tissues were collected and cultured in BSK supplemented with 1× *Borrelia* antibiotic mixture (Sigma). The outgrowth of spirochetes in these cultures was assessed using darkfield microscopy.

### Recombinant BosR expression and purification

Recombinant BosR was produced in *E. coli* using the Champion pET SUMO protein expression system (Invitrogen). Briefly, *bosR* was amplified using primers ZM69F and ZM69R, and ligated into pET SUMO vector through TA cloning such that the resultant construct pOY73 encoded a fusion protein with a His_6_ –SUMO tag at its N terminus. Constructs were confirmed using PCR amplification, restriction digestion, and sequence analysis. The resulting construct, pOY73, was then transformed into *E. coli* strain BL21-DE3. After induction with 1 mM IPTG (Sigma), recombinant His_6_ –SUMO-tagged BosR was purified using a Ni-NTA spin column under native conditions according to the manufacturer's instruction (Qiagen). The N-terminal His_6_ –SUMO tag was removed via cleavage with SUMO protease (Invitrogen) at 30°C for 4 h in the buffer A containing 20 mM Tris, 20 mM NaCl, 100 mM L-arginine, pH 7.5. The protease digestion mixture was concentrated and buffer exchanged with buffer A using an Amicon ultracentrifuge filter device (Millipore) with a 10,000 molecular weight exclusion limit. The concentrated protein was applied to a HiLoad 16/60 Superdex 200 prep grade column and purified on an Äkta fast performance liquid chromatography system (GE Healthcare) using buffer A. Subsequent to elution, peak fractions were analyzed by SDS-PAGE and Western Blot. At this stage, the protein was pure to apparent homogeneity and predominantly present as dimer. Fractions containing pure BosR with a homogeneity >95% were pooled. Protein concentration was determined using the BCA protein assay kit (Thermo Scientific). Rat polyclonal antibody against BosR, Ab-BosR, was generated as previously described [34].

### Metal content analysis

Metal contents in protein or buffer solutions (as references) were measured using inductively coupled plasma atomic emission spectrometry (ICP-AES), by the Research Analytical Laboratory, University of Minnesota. Proteins were demetallated by dialyzing samples with 10 mM ETDA for 24 h, as described previously [49]. Three independent tests were performed, and average metal concentrations with standard deviations were presented.

### SDS-PAGE and immunoblot analysis

SDS-PAGE and immunoblot analysis were carried out as previously described [34]. Briefly, purified protein samples or a volume of whole cell lysate equivalent to 4×10^7^ spirochetes were loaded per lane on a 12.5% acrylamide gel. Resolved proteins were either stained with Coomassie brilliant blue or transferred to nitrocellulose membrane for immunoblot analysis. BosR was detected using the anti-BosR rat polyclonal antibody, Ab-BosR. Rrp2, RpoS, OspC, and DbpA were detected using anti-Rrp2 monoclonal antibody 5B8-100-A1, anti-RpoS monoclonal antibody 6A7-101, anti-OspC monoclonal antibody 1B2-105A, or anti-DbpA monoclonal antibody 6B3, respectively. To confirm equal loading of bacteria in each lane, immunoblotting for the flagellar core protein (FlaB) was performed using a chicken IgY anti-FlaB antibody. Immunoblots were developed colorimetrically using 4-chloro-1-napthol as the substrate or by chemiluminescence using ECL Plus Western Blotting Detection system (Amersham Biosciences).

### Electrophoretic mobility shift assay (EMSA)

Primers used in EMSA are listed in Table S1. PCR-amplified or synthesized DNA probes were end-labeled with digoxigenin using recombinant terminal transferase (Roche Applied Science). The labeled probe (30 fmol) was mixed with various amounts of purified BosR in 20 µl of the gel shift binding buffer containing 20 mM Hepes (pH 7.5), 50 µg/ml poly[d(A-T)], 5% (w/v) glycerol, 1 mM DTT, 100 µg/ml BSA, 1 mM MgCl_2_, and 50 mM KCl. After being incubated at 37°C for 30 min, the samples were analyzed by 5% non-denaturing polyacrylamide gel electrophoresis at 80V for 1–3 h. Then DNA was transferred onto a positively charged Nylon membrane (Roche Applied Science, USA) by electroblotting. The digoxigenin-labeled probes were subsequently detected by an enzyme immunoassay using an antibody (anti-digoxigenin-AP, Fab fragments) and the chemiluminescent substrate disodium 3-(4-methoxyspiro (l,2-dioxetane-3,2′-(5′-chloro)tricyclo[3.3.1.1^3,7^]decan}-4-yl) phenyl phosphate (CSPD) (Roche Applied Science, USA).

### DNase I footprinting assays

For DNase I footprinting, DNA probe (P*rpoS*) containing the *rpoS* promoter was PCR-amplified using primers 88F and 88R. The resultant DNA was labeled with T4 polynucleotide kinase (NEB) and γ-^32^P-ATP (PerkinElmer). A 50-µl reaction containing the radiolabeled probe (300 fmol) and various amounts of BosR was incubated in the gel shift binding buffer at 37°C for 30 min. Then incubated DNA was digested with 0.01 unit of DNase I (Invitrogen) at room temperature for 2 min. The reaction was terminated by adding 100 µl of DNase I stop solution containing 200 mM Tris-HCl (pH 7.5), 50 mM EDTA, 2% SDS, 200 µg/ml of proteinase K, and 250 µg/ml of glycogen, followed by phenol/chloroform extraction. Then DNA was precipitated with 20 µl of 3M ammonium acetate, and 600 µl of 100% ethanol at −20°C overnight. The precipitated DNA was washed with 70% ethanol and air-dried. The pellet was resuspended in 10 µl of formamide dye (90% formamide, 1× TBE, and 0.02% bromophenol blue/xylene cyanol) and analyzed in a 6% polyacrylamide/7 M urea gel at 75 W for 2 h. The gel was transferred onto a chromatography paper (Fisher), dried, and exposed in a PhosphorImager screen. The signals were detected by Typhoon 9200 PhosphorImager (GE Healthcare).

### RNA isolation and RT-PCR

Spirochetes were grown in BSK at 37°C under 5% CO_2_, and harvested when bacterial growth reached a density of 5×10^7^ cells per ml. Total RNA was isolated using Trizol (Invitrogen) according to the instructions. After genomic DNA was digested using RNase-free DNase I (GenHunter Corporation), RNA was further purified using RNeasy Mini Kit (Qiagen). cDNA was generated from 1 µg of RNA using the SuperScript III Platinum Two-step qRT-PCR kit according to the manufacturer's protocol (Invitrogen).

## Supporting Information

Figure S1 Construction of *bosR* mutants (*bosR* ^−^) and complemented strains (*bosR* ^−/+^). (A) Schematic drawings of wild type (WT), the *bosR* mutant, and the complemented strain at the loci of *bb0645-bb0649*. (B) RT-PCR indicate that *bb0646*, *bosR*, and *bb0648* are co-transcribed. lane 1, primer pair a1 and b1 in control PCR using RNA as template; lane 2, primer pair a1 and b1 in ordinary PCR using genomic DNA as template; lanes 3-6, cDNA was used as template; lane 3, primer pair a1 and b1; lane 4, primer pair b3 and b4; lane 5, primer pair b3 and c1; lane 6, primer pair a1 and c1; lane 7, primer pair a1 and c1 using genomic DNA as template. (C) PCR analysis of WT 297, *bosR* mutants, and the complemented strains. The *bosR*-specific primer pairs used in PCR are indicated on the right. Lane 1, WT 297; lane 2, *bosR* ^−^ OY08/A11; lane 3, *bosR* ^−^ OY08/F4; lane 4, *bosR* ^−/+^ OY33/A6; lane 5, *bosR* ^−/+^ OY33/F7. RT-PCR (D) and immunoblot analyses (E) were employed to determine the expression of BosR. α-BosR: rat polyclonal antibody against BosR. Lanes and primer pair designations are as in (C).
(9.61 MB TIF)

Figure S2 Characterization of the *bosR* mutants. (A) Plasmid contents of Bb parental strain 297 (Bb297) and the *bosR* mutant clone OY08/A11 via PCR amplification. Each plasmid for detection is designated above each gel lane. DNA size standards (M) are indicated at the left in base pairs. (B) *In vitro* growth of *bosR* mutants. *Bb* was inoculated into BSK-II medium at 1000 spirochetes/ml and grown at 37°C. Spirochetes were enumerated using dark-field microscopy. Values are the means from three independent experiments. Error bars indicate standard deviations (*n*  =  3). *Bb* strain designations are: WT Bb297; *bosR* mutants OY08/A11 and OY08/F4; complemented strains OY33/A6 and OY33/F7.
(8.88 MB TIF)

Figure S3 3D structural analysis of the Bb BosR protein. The 3D model of BosR was generated using the Swiss-model server (http://swissmodel.expasy.org/) based on the *Vibrio Cholerae* Fur protein structure (Protein Data Bank: 2W57B) as a template. The structure of PerR protein was derived from the active form of PerR from *Bacillus subtilis* (Protein Data Bank: 3F8NA).
(7.75 MB TIF)

Table S1 Oligonucleotide primers used in this study.
(0.03 MB PDF)
