# Supplementary figures and images for: BosR (BB0647) Controls the RpoN-RpoS Regulatory Pathway and Virulence Expression in Borrelia burgdorferi by a Novel DNA-Binding Mechanism
Source: PLoS Pathog. 2011 Feb 10;7(2):e1001272. doi: 10.1371/journal.ppat.1001272 (PMC3037356; doi:10.1371/journal.ppat.1001272)

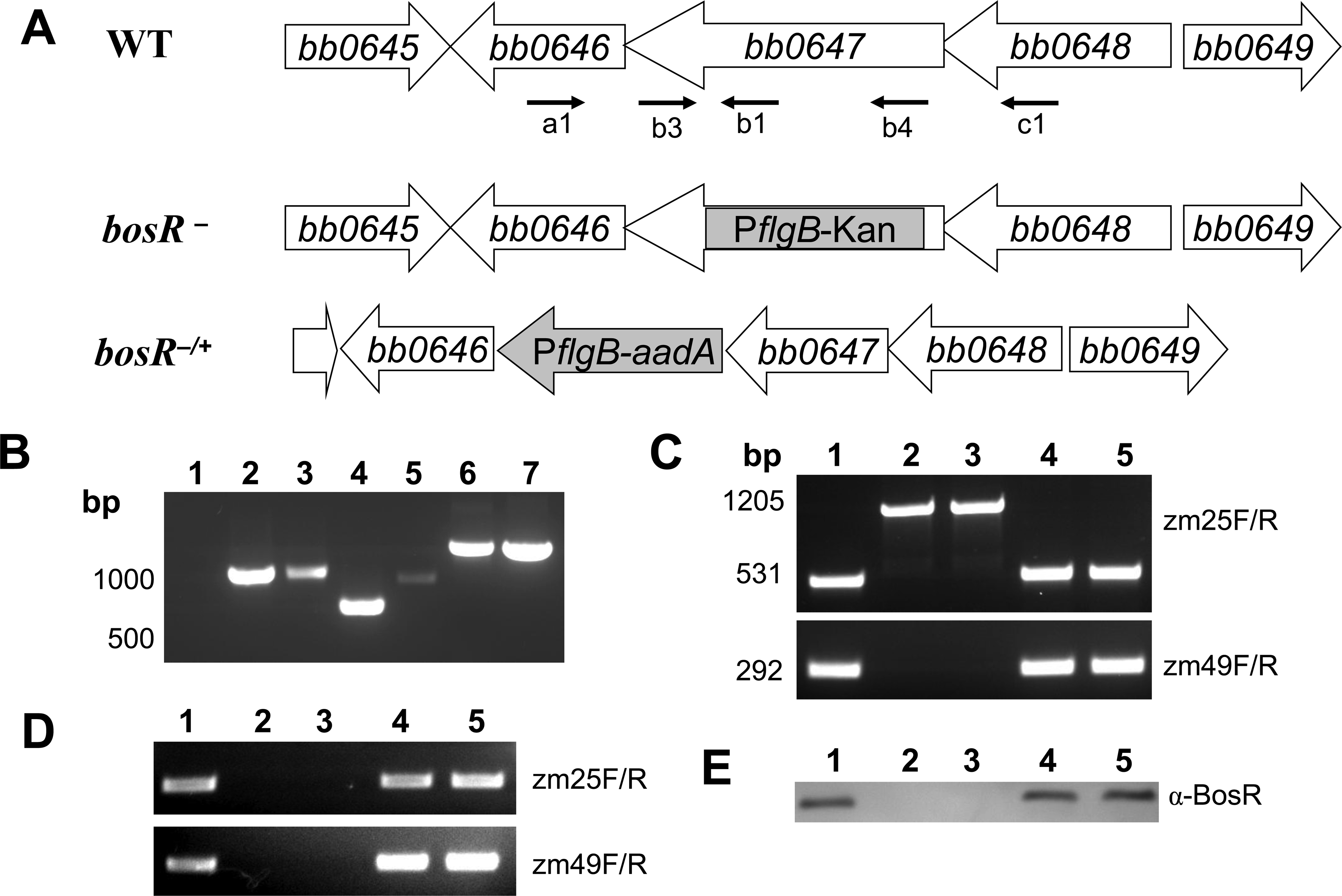

Supplement: Figure S1 — Construction of bosR mutants (bosR −) and complemented strains (bosR −/+). (A) Schematic drawings of wild type (WT), the bosR mutant, and the complemented strain at the loci of bb0645-bb0649. (B) RT-PCR indicate that bb0646, bosR, and bb0648 are co-transcribed. lane 1, primer pair a1 and b1 in control PCR using RNA as template; lane 2, primer pair a1 and b1 in ordinary PCR using genomic DNA as template; lanes 3-6, cDNA was used as template; lane 3, primer pair a1 and b1; lane 4, primer pair b3 and b4; lane 5, primer pair b3 and c1; lane 6, primer pair a1 and c1; lane 7, primer pair a1 and c1 using genomic DNA as template. (C) PCR analysis of WT 297, bosR mutants, and the complemented strains. The bosR-specific primer pairs used in PCR are indicated on the right. Lane 1, WT 297; lane 2, bosR − OY08/A11; lane 3, bosR − OY08/F4; lane 4, bosR −/+ OY33/A6; lane 5, bosR −/+ OY33/F7. RT-PCR (D) and immunoblot analyses (E) were employed to determine the expression of BosR. α-BosR: rat polyclonal antibody against BosR. Lanes and primer pair designations are as in (C). (9.61 MB TIF) [file ppat.1001272.s001.tif]

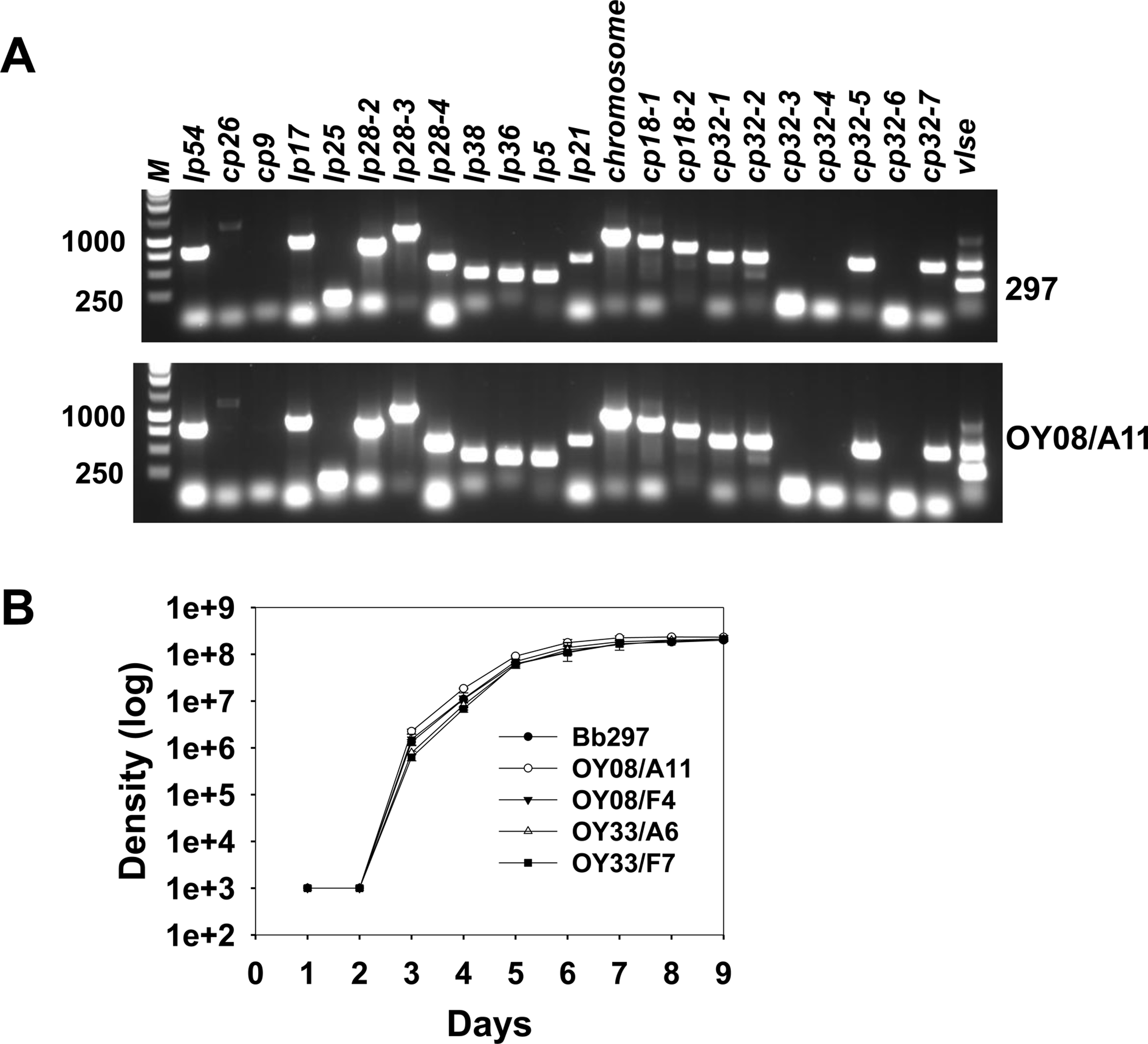

Supplement: Figure S2 — Characterization of the bosR mutants. (A) Plasmid contents of Bb parental strain 297 (Bb297) and the bosR mutant clone OY08/A11 via PCR amplification. Each plasmid for detection is designated above each gel lane. DNA size standards (M) are indicated at the left in base pairs. (B) In vitro growth of bosR mutants. Bb was inoculated into BSK-II medium at 1000 spirochetes/ml and grown at 37°C. Spirochetes were enumerated using dark-field microscopy. Values are the means from three independent experiments. Error bars indicate standard deviations (n = 3). Bb strain designations are: WT Bb297; bosR mutants OY08/A11 and OY08/F4; complemented strains OY33/A6 and OY33/F7. (8.88 MB TIF) [file ppat.1001272.s002.tif]

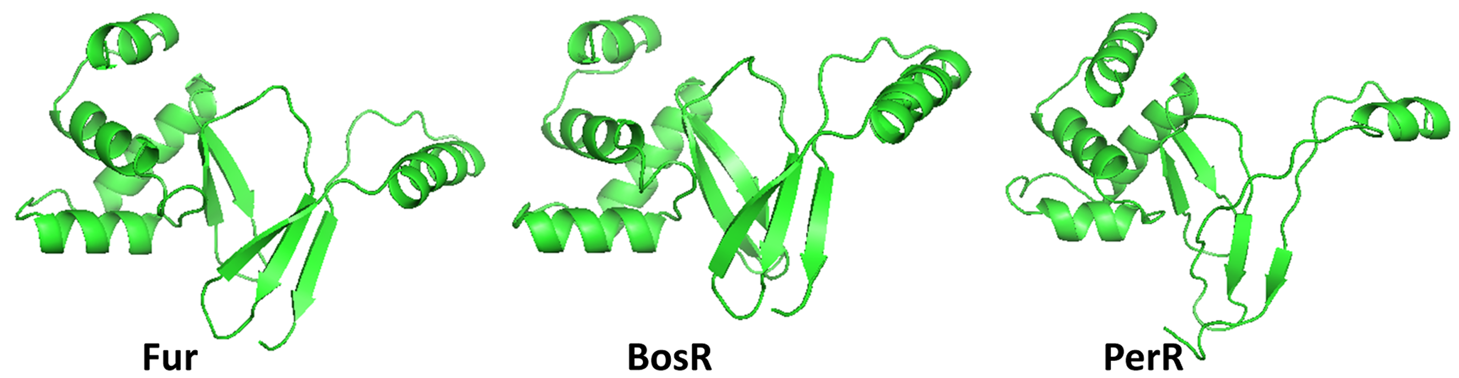

Supplement: Figure S3 — 3D structural analysis of the Bb BosR protein. The 3D model of BosR was generated using the Swiss-model server (http://swissmodel.expasy.org/) based on the Vibrio Cholerae Fur protein structure (Protein Data Bank: 2W57B) as a template. The structure of PerR protein was derived from the active form of PerR from Bacillus subtilis (Protein Data Bank: 3F8NA). (7.75 MB TIF) [file ppat.1001272.s003.tif]
