# Supplementary material for: BosR (BB0647) Controls the RpoN-RpoS Regulatory Pathway and Virulence Expression in Borrelia burgdorferi by a Novel DNA-Binding Mechanism
Source: PLoS Pathog. 2011 Feb 10;7(2):e1001272. doi: 10.1371/journal.ppat.1001272 (PMC3037356; doi:10.1371/journal.ppat.1001272)
Supplement: Table S1 — Oligonucleotide primers used in this study. (0.03 MB PDF) [file ppat.1001272.s004.pdf]

**Table S1.** Oligonucleotide primers used in this study

| Primer | Sequence (5'-3') |
|---|---|
| **General PCR or cloning** | |
| ZM24F, a1 | AGCCTCATCAATGTCTCTCAGCCA |
| ZM25.2F, c1 | ATGGATCCGGGTTCTTATAACACAGTTAAGATCCAAG |
| ZM25F, b4 | CGGAATTCATGAACGACAACATAATAGACGTA |
| ZM25R, b3 | ATGGATCCTCATAAAGTGATTTCCTTGTTCTCAT |
| ZM49F, b1 | GTCGGCATTACAAACGATCCTG |
| ZM49R | AGTGAGCTATTGTGGAAGCCAAGC |
| bb0646F | TCTTGAAAATTCACACGCTCACTT |
| bb0646R | TGTGTTTTGGGCGCTTTTG |
| 297 ospC-66F | AGGAAAAGATGGGAATACATC |
| 297 ospC-288R | TGTTCCATTATGCCCCGC |
| ZM009F | GTTTGTTGCAGCCAGAAGCCTGAT |
| ZM009R | TCTAACAGCCCACGGAACAGTTGA |
| ZM111F | CCCAACGCTCGAATTTAAAGACCC |
| ZM111R | TGCTCCACCAACAGAGCTAA |
| ZM014F | GGGAACTTGATTAGCCTGCGCAAT |
| ZM014R | TCGAGCTTCTGATGATGCTGCT |
| ZM015F | CTTGCAGGACAAATACAAAGAGGC |
| ZM015R | TGGGACTATTGTCCAGGTTATATCTTT |
| bba24-204 | GGGTAGTGGGGTATCAGAAAATC |
| bba24-451 | GAGCTGTAGTTGGAGGATTCTC |
| ZM88F | TATCCATGGTGCTAAACGGAGGCCAAGTAGAAG |
| ZM88.2F | TATCCATGGCACAGTTTTTGCATGAAAAT |
| ZM88.2R | GGGCCCATATGAGCAGCTCTTATTAATCCCAAGTTGCC |
| 97F | TATCCATGGTCTTTGCATTGGGGTTGTG |
| 97R | GGGCCCATATGTATTGCGTCTAAATCATCCTT |

| | |
|---|---|
| ZM107F | TATCCATGGCCTGAGTATTCATTATATAAGTC |
| ZM108R | GCGCATATGTAATTTGTGCCTCCTTTTTATTTATG |
| ZM57 | AGATCTATTTTATTTTATTTTTCATAAAGTGGGCTAAA |
| ZM61 | CATATGCTTTTCCCGTGGCTTCTTTT |
| ZM69F | ATGAACGACAACATAATAGACGTA |
| ZM69R | TCATAAAGTGATTTCCTTGTTCTCAT |
| **For EMSA** | |
| ZM126F | TTGCATGGAAATTAAGTAGTAAAAACTTAATCACAATATTCAAGAAAGGGGAGAAAATAT |
| ZM126R | ATATTTTCTCCCCTTTCTTGAATATTGTGATTAAGTTTTTACTACTTAATTTCCATGCAA |
| ZM127F | TTTAGTTTAAAACCATTTTTAAATTAAATTGGCACAGTTTTTGCATGAAAATTAAGTAGT |
| ZM127R | ACTACTTAATTTTCATGCAAAAACTGTGCCAATTTAATTTAAAAATGGTTTTAAACTAAA |
| ZM132F | GGCCTTGCCGATTTAATTTACAATCAATTACAAAAAAGTAAATAATTCAAAAAATACTCC |
| ZM132R | GGAGTATTTTTTGAATTATTTACTTTTTTGTAATTGATTGTAAATTAAATCGGCAAGGCC |
| ZM147F | AAAAAGCTGAGATCTGTCGACATCCATGGTGGCACAGTTTTTGCATGAAAATTAAGTAGT |
| ZM147R | ACTACTTAATTTTCATGCAAAAACTGTGCCACCATGGATGTCGACAGATCTCAGCTTTTT |
| ZM149F | TTTAGTTTAAAACCATTTTTAAATTAAATTGGCACAGTTTTTGCATTTTAACAGGCAAAA |
| ZM149R | TTTTGCCTGTTAAAATGCAAAAACTGTGCCAATTTAATTTAAAAATGGTTTTAAACTAAA |
| ZM157F | TTTAGTTTAAAACCATTTTTAAATTAAATTTTCACAGTTTTTGAATTCCAACAGGCAAAA |
| ZM157R | TTTTGCCTGTTGGAATTCAAAAACTGTGAAAATTTAATTTAAAAATGGTTTTAAACTAAA |
| ZM160F | TCACAATATTCAAGAAAGGGGAGAAAATATAATAACTATGAACATATTTAGTAATGAGGA |
| ZM160R | TCCTCATTACTAAATATGTTCATAGTTATTATATTTTCTCCCCTTTCTTGAATATTGTGA |
| ZM161F | AACATATTTAGTAATGAGGATTTAAACATATATTTAAAATCAGTAAGAGAACACAAGCTA |
| ZM161R | TAGCTTGTGTTCTCTTACTGATTTTAAATATATGTTTAAATCCTCATTACTAAATATGTT |
| ZM166F | TTTAGTTTAAAACCATTTTTAGGCCAAATTTTCACAGTTTTTGAATTCCAACAGGCAAAA |
| ZM166R | TTTTGCCTGTTGGAATTCAAAAACTGTGAAAATTTGGCCTAAAAATGGTTTTAAACTAAA |
| ZM155F | CTCAAAGCTTTGGCCTTGCCGATTTAATTTACAATCAATTACAAAGCTGAGATCTGTCGA |
| ZM155R | TCGACAGATCTCAGCTTTGTAATTGATTGTAAATTAAATCGGCAAGGCCAAAGCTTTGAG |
| ZM156F | GCTGAGATCTGTCGAATCAATTACAAAAAAGTAAATAATTCAAAAAATACTCCCCCTAAA |

| | |
|---|---|
| ZM156R | TTTAGGGGGAGTATTTTTTGAATTATTTACTTTTTTGTAATTGATTCGACAGATCTCAGC |
| ZM210F | CTCAAAGCTTTGGCCTTGCCGATTTGGCCTACAATCAATTACAAAGCTGAGATCTGTCGA |
| ZM210R | TCGACAGATCTCAGCTTTGTAATTGATTGTAGGCCAAATCGGCAAGGCCAAAGCTTTGAG |
| ZM211F | GCTGAGATCTGTCGAATCAATTACAAAAAAGTAGGCCATTCAAAAAATACTCCCCCTAAA |
| ZM211R | TTTAGGGGGAGTATTTTTTGAATGGCCTACTTTTTTGTAATTGATTCGACAGATCTCAGC |
| ZM212F | ATTTTATATCCTATTTAGTTTAAAACCATTTTTAAATTAAATTGGCACAGTTTTTGCATG |
| ZM212R | CATGCAAAAACTGTGCCAATTTAATTTAAAAATGGTTTTAAACTAAATAGGATATAAAAT |
| ZM213F | ATTTTATATCCTAGGCCGTTTAAAACCATTTTTAAATTAAATTGGCACAGTTTTTGCATG |
| ZM213R | CATGCAAAAACTGTGCCAATTTAATTTAAAAATGGTTTTAAACGGCCTAGGATATAAAAT |
| ZM214F | ATTTTATATCCTATTTAGTTTAAAACCATTTTTAGGCCAAATTGGCACAGTTTTTGCATG |
| ZM214R | CATGCAAAAACTGTGCCAATTTGGCCTAAAAATGGTTTTAAACTAAATAGGATATAAAAT |
| ZM215F | TGGTTTAGATTTAATTTTTTTCTCAATTCAGCAATTTCTGAATATAGAATTTTCATATCT |
| ZM215R | AGATATGAAAATTCTATATTCAGAAATTGCTGAATTGAGAAAAAAATTAAATCTAAACCA |
| ZM216F | TGGTTTAGATGGCCTTTTTTTCTCAATTCAGCAATTTCTGAATATAGAATTTTCATATCT |
| ZM216R | AGATATGAAAATTCTATATTCAGAAATTGCTGAATTGAGAAAAAAAGGCCATCTAAACCA |
| ZM217F | AGTGTAAAAATTATAATCAATTTTTTCATTAAATATCCTTTTTATTTATTATGGTATTAA |
| ZM217R | TTAATACCATAATAAATAAAAAGGATATTTAATGAAAAAATTGATTATAATTTTTACACT |
| ZM218F | AGTGTAAAAAGGCCAATCAATTTTTTCATTAAATATCCTTTTTATTTATTATGGTATTAA |
| ZM218R | TTAATACCATAATAAATAAAAAGGATATTTAATGAAAAAATTGATTGGCCTTTTTACACT |
| ZM223F | ATTTTATATCCTAGGCCGTTTAAAACCATTTTTAGGCCAAATTGGCACAGTTTTTGCATG |
| ZM223R | CATGCAAAAACTGTGCCAATTTGGCCTAAAAATGGTTTTAAACGGCCTAGGATATAAAAT |
| ZM219F | AAAAAAATATGAAAATAAATAAATAATAAGTAGTAAAATATTAATAACTGGGTATAAAAT |
| ZM219R | ATTTTATACCCAGTTATTAATATTTTACTACTTATTATTTATTTATTTTCATATTTTTTT |
| ZM220F | AAAAAAATATGAAAATGGCCAAATAATAAGTAGTAAAATATTAATAACTGGGTATAAAAT |
| ZM220R | ATTTTATACCCAGTTATTAATATTTTACTACTTATTATTTGGCCATTTTCATATTTTTTT |
